# NF-κB over-activation portends improved outcomes in HPV-associated head and neck cancer

**DOI:** 10.18632/oncotarget.28232

**Published:** 2022-05-24

**Authors:** Travis P. Schrank, Andrew C. Prince, Tejas Sathe, Xiaowei Wang, Xinyi Liu, Damir T. Alzhanov, Barbara Burtness, Albert S. Baldwin, Wendell G. Yarbrough, Natalia Issaeva

**Affiliations:** ^1^Department of Otolaryngology/Head and Neck Surgery, UNC, Chapel Hill, NC 27599, USA; ^2^Department of Surgery, Otolaryngology, Yale, New Haven, CT 06519, USA; ^3^Current address: Department of Surgery, Columbia University, New York, NY 10032, USA; ^4^Department of Pharmacology and Bioengineering, University of Illinois at Chicago, Chicago, IL 60612, USA; ^5^Bioinformatics Core, University of Illinois Cancer Center, Chicago, IL 60612, USA; ^6^Department of Medicine, Yale School of Medicine, New Haven, CT 06510, USA; ^7^Lineberger Cancer Center, UNC, Chapel Hill, NC 27599, USA; ^8^Department of Pathology and Laboratory Medicine, UNC, Chapel Hill, NC 27599, USA; ^9^Yale Cancer Center, Yale School of Medicine, New Haven, CT 06510, USA; ^*^These authors contributed equally to this work; ^#^Senior authors

**Keywords:** HPV, head and neck cancer, CYLD, TRAF3, NF-κB

## Abstract

Evolving understanding of head and neck squamous cell carcinoma (HNSCC) is leading to more specific diagnostic disease classifications. Among HNSCC caused by the human papilloma virus (HPV), tumors harboring defects in *TRAF3 or CYLD* are associated with improved clinical outcomes and maintenance of episomal HPV. TRAF3 and CYLD are negative regulators of NF-κB and inactivating mutations of either leads to NF-κB overactivity. Here, we developed and validated a gene expression classifier separating HPV+ HNSCCs based on NF-κB activity. As expected, the novel classifier is strongly enriched in NF-κB targets leading us to name it the NF-κB Activity Classifier (NAC). High NF-κB activity correlated with improved survival in two independent cohorts. Using NAC, tumors with high NF-κB activity but lacking defects in *TRAF3* or *CYLD* were identified; thus, while *TRAF3 or CYLD* gene defects identify the majority of tumors with NF-κB activation, unknown mechanisms leading to NF-kB activity also exist. The NAC correctly classified the functional consequences of two novel *CYLD* missense mutations. Using a reporter assay, we tested these CYLD mutations revealing that their activity to inhibit NF-kB was equivalent to the wild-type protein. Future applications of the NF-κB Activity Classifier may be to identify HPV+ HNSCC patients with better or worse survival with implications for treatment strategies.

## INTRODUCTION

Head and neck squamous cell carcinoma (HNSCC) is a devastating disease that impairs fundamental tissues involved in respiration, phonation, and digestion. It is categorized into two discrete diseases based on etiology: human papillomavirus (HPV) negative HNSCC, which is primarily caused by exposure to ethanol and tobacco, and HPV-associated (HPV+) HNSCC [1]. These forms of HNSCC have contrasting clinical, epidemiological, and histological features [2–4] with HPV+ HNSCC occurring in a younger population with less or no smoking history [5, 6]. HPV-mediated cancer arises primarily in the reticulated epithelia of the oropharynx (e.g., tonsils, base of tongue), whereas HPV-negative HNSCC is found at all subsites (e.g., oral cavity, larynx) [2]. Unfortunately, the global incidence of HPV+ HNSCC is increasing, and for at least a decade, HPV has caused more head and neck cancers than uterine cervical cancers annually in the United States [7, 8].

Since HPV+ HNSCC has only recently been recognized as a distinct clinicopathological entity [9], management of HNSCC has been driven by escalating therapies to improve cancer control in the more treatment-resistant HPV-negative HNSCC [2, 6]. While oncologic outcomes for HPV+ HNSCC are generally favorable, treatment paradigms developed for HPV-negative disease burden many survivors of HPV+ HNSCC with lifelong debilitating treatment-associated side effects [10]. On the other hand, ~30% of HPV+ HNSCC patients exhibit a more aggressive disease course and suffer recurrence [11, 12]. Therefore, there is a growing clinical demand to develop robust stratification tools to accurately identify patients with good or poor prognosis and that could be used to personalize treatment.

TRAF3 belongs to the TRAF family of proteins that are known as intracellular adaptors and E3 ubiquitin ligases mediating receptor-based signaling [13]. TRAF3 polyubiquitinates and degrades NF-κB-inducing kinase (NIK) restraining non-canonical NF-κB signaling. The deubiquitinating enzyme Cylindromatosis (CYLD) is a tumor suppressor that was found to be mutated in familiar cylindromatosis, a condition associated with benign skin tumors. CYLD mediates deubiquitination of the NF-κB essential modulator (NEMO) thus inhibiting canonical NF-κB signaling [14, 15]. A cross talk between canonical and non-canonical NF-κB signaling suggests that TRAF3 and CYLD affect both NF-κB pathways.

Somatic defects in the NF-κB inhibitors *TRAF3* and *CYLD* are found in ~30% of HPV+ HNSCC tumors [1, 16, 17]. These gene defects are uncommon in uterine cervical cancer and HPV-negative HNSCC. While frequent *TRAF3* or *CYLD* inactivating mutations are found in B cell lymphomas, where constitutive NF-κB activity is known to play a key survival role [18–20], these mutations are rarely found in solid tumors [16]. Exceptions with more frequent *TRAF3* and *CYLD* mutations include two virally-associated cancers, HPV+ HNSCC and Epstein-Barr virus-associated nasopharyngeal carcinoma (NPC) [21–23]. Although initial studies focused on NF-κB activity as a defense against viral infections, further investigation revealed more nuances with some viruses, like EBV and HIV, depending on NF-κB activity to support viral replication and viral gene expression [24–27]. The correlation between TRAF3 and CYLD alterations and the lack of classic oncogenic HPV integration events, suggests that HPV may similarly exploit NF-κB activity in HNSCC to be able to maintain extrachromosomal HPV genomic material.

The power of multi-variable models and/or multi-omic approaches can be harnessed to improve tumor subtyping [28–31]. For example, an RNA expression-based PARP inhibitor outcome prediction model in ovarian cancer outperformed BRCA1/2 mutational status in predicting treatment response [30]. In the present study, transcriptional differences between tumors with and without *TRAF3* and *CYLD* defects formed the basis for a novel classification of HPV+ HNSCC. Based on established roles of *TRAF3* and *CYLD* as inhibitors of NF-κB, it was expected that the resultant classifier would segregate tumors on the basis of NF-κB activity. Gene set enrichment analysis confirmed that the classifier identified tumors with high or low NF-κB activity and, relative to TRAF3 and CYLD defects, this NF-κB Activity Classifier (NAC) improved identification of tumors with good and poor survival. Among TCGA specimens, two novel missense mutations in *CYLD* were identified: N300S and D618A [16]. To understand the implications of these point mutations, we used the NAC and correlated results with a cell-based assay to evaluate their effect on NF-κB transcriptional activity.

To improve on genomic classification, we designed this study to provide a foundation for development of NF-κB related, RNA based classification strategies to better identify HPV+ HNSCC patients with good or poor prognosis that could potentially aid in future efforts towards treatment personalization.

## RESULTS

### Development of the NF-κB activity classifier (NAC)

**Figure 1:**
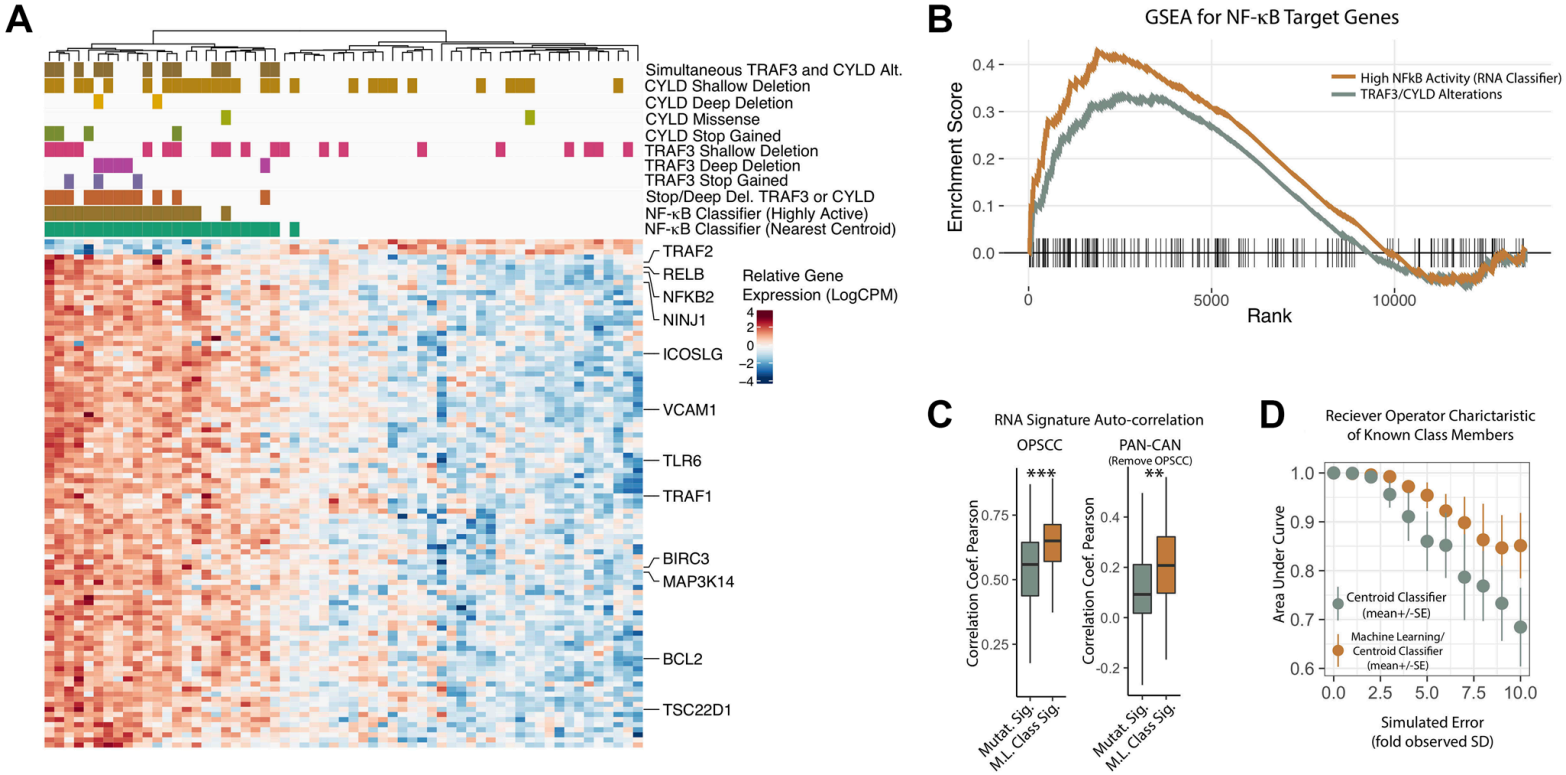
Development of an NF-κB activity related RNA expression classifier. (**A**) Heatmap of RNA Expression Changes Associated with TRAF3/CYLD Alterations and Deletions. Normalized log_2_(read counts per million), color scaled by row. Columns– Tumor Samples, organized by unguided clustering. Rows – Top 100 genes by *p*-value differentially expressed between high-confidence NF-κB active vs. inactive tumors (see methods for details). Row annotation – Known NF-κB target genes curated from literature review. Column Annotation Details: Track 1 (green) - RNA classifier (“NF-κB active”) based on nearest centroid. Track 2 (green brown) - RNA classifier (“NF-κB highly active”) based on minimal classifier score identified for TRAF3/CYLD nonsense or frameshift mutation bearing tumors. Track 3 (orange) – Tumor contains a frameshift, nonsense, or deep deletion in TRAF3 or CYLD. Track 4 (purple) - Tumor contains a frameshift or nonsense mutation in TRAF3. Track 5 (lavender) - Tumor contains a deep deletion in TRAF3. Track 6 (pink) - Tumor contains a shallow deletion in TRAF3. Track 7 (army green) - Tumor contains a frameshift or nonsense mutation in CYLD. Track 8 (lime green) - Tumor contains a missense mutation in CYLD. Track 9 (yellow) - Tumor contains a deep deletion in CYLD. Track 10 (mustard) - Tumor contains a shallow deletion in CYLD. Track 11 (dark brown) – Tumor contains any alteration in both TRAF3 and CYLD. Shallow Deletion – Gistic copy-number score = −1, Deep Deletion – Gistic copy-number score = −2, Stop Gained – frameshift or nonsense mutation. Missense – missense or in frame indel. Stop/Deep Del. – Any one of nonsense, frameshift, or deep deletion. (**B**) Gene Set Enrichment Analysis for NF-κB Target Genes. All available genes after data filtering (see methods) were ranked according to signal-to-noise ratio when comparing the two groups of tumors. The MiSigDB Hallmark TNFA/NFkB gene set was tested for enrichment. NF-κB highly active – tumors were defined according to RNA based classifications (see methods); these were compared to all other tumors in the study cohort. NF-κB Pathway Alteration – Any missense, nonsense, frameshift, deep deletion in TRAF3 and/or CYLD; these were compared to all other tumors in the study cohort. Lines – enrichment score values. Dashed Line – maximum achieved enrichment score (NFkB high activity only). Vertical Hashes – rank positions of the test gene set (Hallmark NF-κB). (**C**) Auto-correlation of RNA Gene Set before and after the machine learning (ML) procedure. (**D**) Classifier Performance of Gene Sets before and after ML improvement, with increasing (simulated) error of measurement. Performance determined by area under the receiver operating characteristic curve. ^***^ *P* value < 5 × 10^−4^, ^**^ *P* value < 5 × 10^−3^.

We previously reported that TRAF3 and CYLD alterations in a subset of HPV+ oropharyngeal squamous cell carcinoma (OPSCC) tumors correlated with NF-κB activation and improved survival [16]. NF-*κB* is a family of inducible transcription factors that play a role in innate and adaptive immune response; constitutively active *NF-κB* is a well-known oncogene in various cancer types that increases cell proliferation, migration, invasion and metastasis while inhibiting apoptosis [32–34]. NF-κB activation, induced by carcinogens or oncogenic viruses, was found in head and neck tumors and cells [35]. Given the variable role that NF-κB plays in HPV+ OPSCC tumorigenesis, we hypothesized that tumor groups based on NF-κB related gene expression may correlate with treatment outcome, considering that tumors lacking defects in *TRAF3* and *CYLD* may have unrecognized mechanisms driving constitutive NF-κB activation. TCGA expression data were first grouped by the presence of a known *TRAF3* or *CYLD* defect and the top 100 differentially expressed genes identified (Figure 1A). As anticipated, gene set enrichment analyses demonstrated a high enrichment score (>0.3) for NF-κB target genes (Figure 1B, Grey) and several notable NF-κB target genes were differentially expressed – *TRAF2, NF-kB2, BIRC3*, and *MAP3K14*.

Machine learning techniques were used to refine the signature resulting in a set of 50 key genes dubbed the NF-κB Activity Classifier Gene Signature (Supplementary Table 1). Using the NF-κB Activity Classifier (nearest centroid), all tumors were then given a final classification to identify tumors with high NF-κB activity (Figure 1, track 1, green). As may be expected based on unknown mechanisms of NF-kB activation, some additional samples without inactivating alterations (deep deletion, nonsense/frameshift mutation) in either *TRAF3* or *CYLD* (Figure 1A, track 3 – burnt orange) were included in the NF-κB active group.

In order to identify a set of tumors with equivalently high activation of NF-κB, as observed with destructive nonsense or frameshift mutations in *TRAF3* or *CYLD*, we also defined a more stringent threshold of NF-κB activation, based on the lowest classifier score observed for the highest confidence destructive alterations (nonsense or frameshift) of *TRAF3* or *CYLD* (see Figure 1, track 2 – green brown). Notably, 6 tumors included in this “highly active” NF-κB group also were found to be without deep deletion, frameshift/nonsense mutation of *TRAF3* or *CYLD,* bolstering the utility of an RNA based approach to identify NF-κB activated HPV+ HNSCC tumors.

All tumors harboring concurrent alterations (including shallow deletions) in both *TRAF3* and *CYLD* were found to be in the NF-κB active group (Figure 1A, track 11 - brown), and two of these tumors were included in the “highly active” NF-κB group. These data suggest an intriguing hypothesis that combinations of more subtle changes simultaneously effecting both *TRAF3* and *CYLD* might also contribute to NF-κB activation.

### RNA-based classification strengthens the association with NF-κB target gene expression

To determine if the NF-κB Activity Classifier enhanced correlation with NF-κB target genes relative to groupings based on *TRAF3/CYLD* alterations, we performed gene set enrichment analysis using *TRAF3/CYLD* (missense, nonsense, frame shift) and the highly active NF-κB classification as determined by the NAC. This analysis demonstrated significant enrichment for the Hallmark NF-κB target gene set for both TRAF3/CYLD and highly active NF-κB classifiers (*p*-value < 0.01); however, stratification using the NF-κB Activity Classifier demonstrated stronger enrichment (Figure 1B).

### Machine learning (ML) improves NF-κB gene set properties and classifier robustness

Auto-correlation, or compactness, is a desirable feature of RNA expression signatures since loss of compactness when applied to new datasets can limit their diagnostic utility [36]. To begin determining compactness of the NF-κB signature auto-correlation was examined. Pearson correlation coefficients were improved after the machine learning procedure, both in the HNSCC tumors used for deriving the gene set; as well as across all tumor types included in the TCGA pan-cancer atlas (Figure 1C). Since clinical expression datasets might be expected to have more error compared to TCGA, we also considered how robust our classifications were to increasing noise of measurement. To examine this, we calculated the area under the receiver-operator characteristic curve (AUC) for the original and ML improved classifier with increasing levels of (random) simulated error applied to the RNA expression data. The ML-improved classifier had higher AUC values at higher levels of noise, and maintained a median AUC of >0.95 even with a five-fold increase in error as compared to the original RNA data from TCGA (Figure 1D). Taken together these analyses illustrate the favorable properties of our NF-κB signature, as well as a high-degree of robustness of the nearest centroid classifications based on these genes.

### Weighted gene correlation network analysis identifies an NF-κB associated gene expression module in HPV+ HNSCC

**Figure 2:**
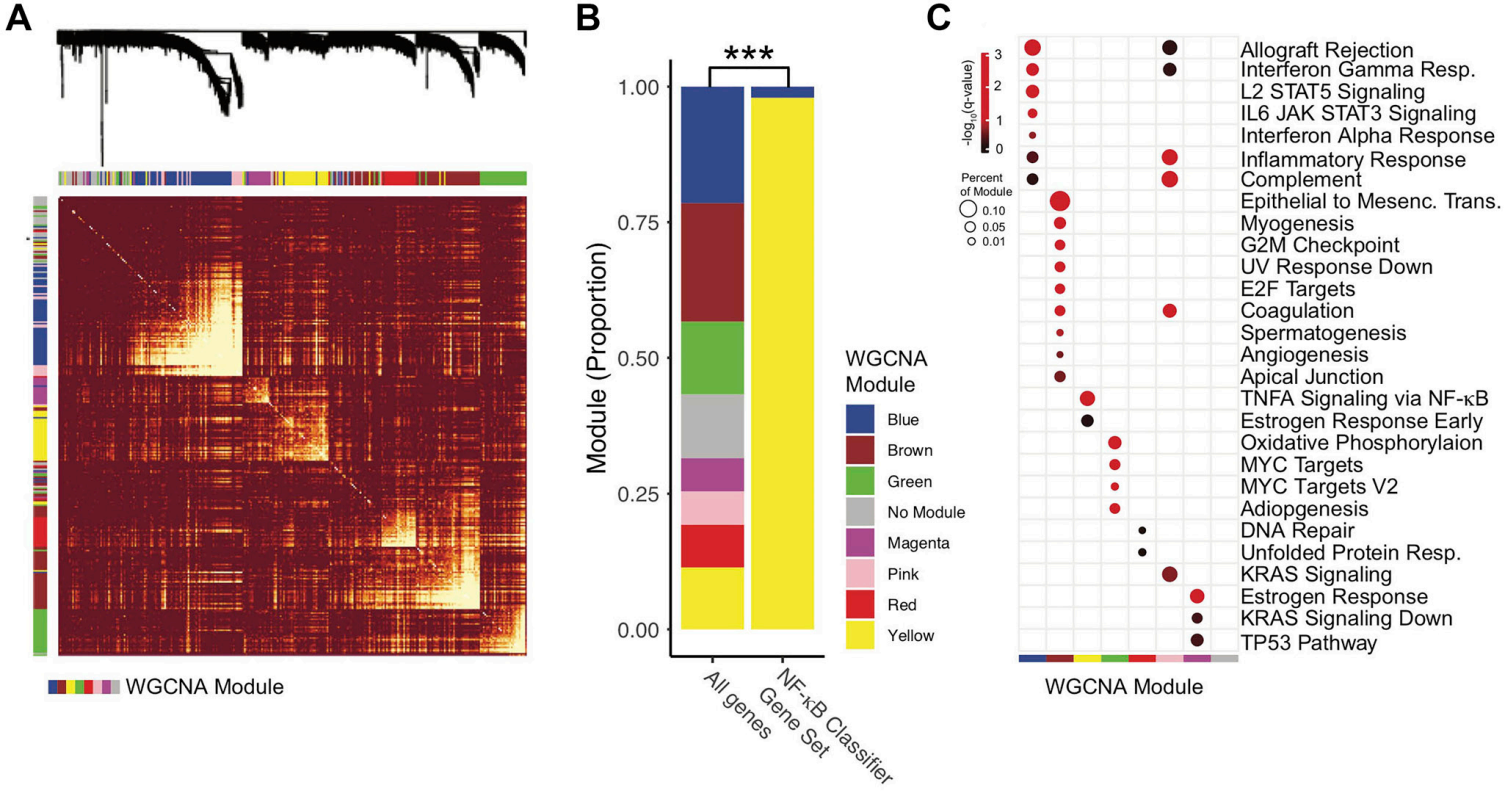
Characterization of the NF-κB activity classifier genes with weighted gene correlation network analysis (WGCNA). Only modules with more than 250 and less than 5000 genes were analyzed. (**A**) Expression Dissimilarity matrix with clustering dendrogram. For clarity, a subset of 1500 genes are displayed. Warmer colors (red) represent higher degrees of dissimilarity. Row and Column Annotations – WGCNA gene expression modules, colors correspond to module name, as in panel C. (**B**) Proportion of Genes by WGCNA module. NF-κB Classifier Gene Set – Gene set (50 genes) used in the NF-κB activity classifier. All genes – Genes analyzed by WGCNA but not included in the NF-κB activity classifier. *P*-value represent chi-squared test. ^***^ *p*-value < 0.0001. (**C**) Hypergeometric Enrichment Plot. Identified WGCNA modules were screened for enrichment in Hallmark Gene Sets from MiSigDB. Red-black color scale indicates adjusted *p*-value (-Log_10_[*q*-value]). Only results with *q* < 0.05 were displayed. Percent of module genes in Hallmark gene set is represented by point size. *Q*-values represent hypergeometric enrichment as reported by the EnrichR R package.

As TRAF3 and CYLD have other molecular functions in addition to inhibiting NF-κB, and to determine the relationship of the NAC to other biological aspects, we performed weighted gene correlation network analysis (WGCNA). To render required processor times tractable, only the 13,000 most highly expressed genes were included in the WGCNA analysis, excluding 2 of the 50 classifier genes. This unguided discovery approach identified 7 sets (or modules) of highly autocorrelated genes; the relative size and correlative dissimilarity between the modules are displayed in Figure 2A. These modules were then screened for (hypergeometric) enrichment of the established hallmark gene sets from the MiSig database (Figure 2C). Interestingly, one module (“yellow”) was found to be most associated with NF-κB target gene expression by both *p*-value and fraction of module genes in the test signature (Figure 2C). Of note, no other modules were enhanced for NF-κB targets. Furthermore, 47 of 48 signature genes included in the WGCNA analysis were found to be in the “yellow” module (Figure 2B, Supplementary Table 2 for WGCNA modules, and Supplementary Table 3 for hypergeometric enrichment analysis). The “yellow” module was also associated with early estrogen receptor signaling (Figure 2C).

### Expression-based classification improves correlation with survival

**Figure 3:**
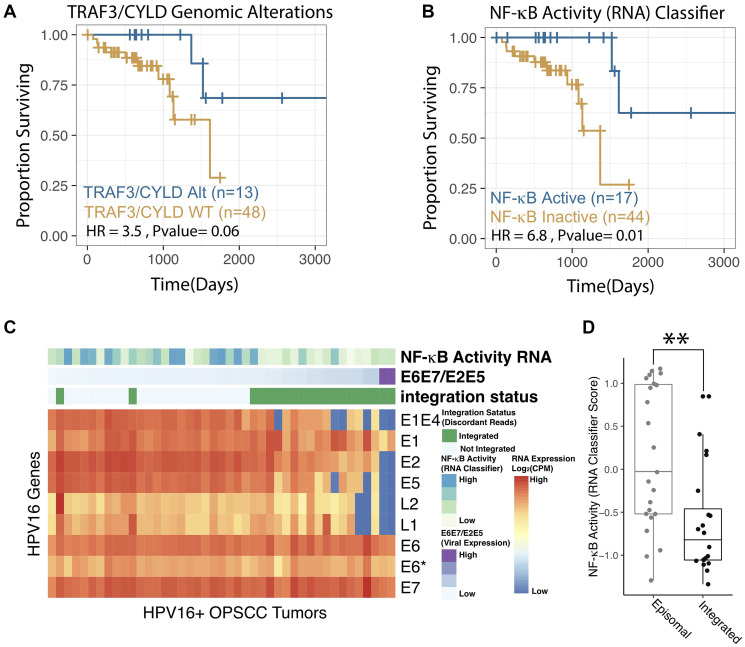
NF-κB activity classifier correlates with patient outcomes and viral integration status. (**A**–**B**) Kaplan-Meier Analysis of Progression Free Interval of HPV+ HNSCC. *P*-values represent log-rank test. HR – Hazard Ratio. NF-κB Active – Highly NF-κB active tumors by RNA expression as defined according to the RNA based classifier (see methods), these were compared to all other tumors (NF-κB Inactive) in the study cohort. TRAF3/CYLD Alt – Any missense, nonsense, frameshift, deep deletion in TRAF3 and/or CYLD, these were compared to all other tumors (TRAF3/CYLD WT) in the study cohort. See Supplementary Figure 1 for grossly similar recurrence-free survival results. (**C**) Heatmap of HPV16 Viral Gene Expression for 61 HPV16+ OPSCC tumors included in the TCGA. Columns – tumors. Rows – HPV16 viral genes. Column Annotations: NF-κB activity RNA - nearest classifier score, higher values are more proximal to the NF-κB active centroid. E6E7/E2E5 Ratio – [E6 expression(raw counts) + E7 expression (raw counts)]/[E2 expression (raw counts) + E5 expression (raw counts)]. The columns are organized by this metric which is reported to strongly correlated with viral genomic integration. Integration Status – HPV viral integration status as determined by the ViFi pipeline. (**D**) Box Plot comparing NF-κB activity in integrated and episomal tumor groups. Integration as assigned by ViFi. NF-κB activity – Raw NF-κB classifier scores as in panel C. ^**^ *p* < 0.001.

Clinical outcomes for the TCGA HPV+ HNSCC cohort were assessed with PFI, available for all TCGA samples [37]. Kaplan-Meier survival curves were created for samples stratified by the presence of a *TRAF3* or *CYLD* genomic alteration (Figure 3A) and using the NF-κB Activity Classifier (Figure 3B). In both cases, a survival advantage was apparent for this distinct disease phenotype. However, the NF-κB Activity Classifier was associated with a larger hazard ratio (HR = 6.8) and statistically significant difference in PFI (*p* = 0.01) (Figure 3A, 3B). Although fewer tumors (*n* = 57) were annotated for recurrence-free survival (RFS), classification of NF-κB active tumors using the NAC also correlated with improved RFS (Supplementary Figure 1, *p*-value = 0.006).

### NF-κB activity correlates with HPV viral integration status

We previously reported that somatic alterations in *TRAF3* and *CYLD* were associated with lack of viral integration in HPV+ HNSCC. To examine if our RNA-based estimates of NF-κB activity also correlated with viral integration, we first determined integration based on discordant read pair mapping - sequences that mapped to both the human and HPV viral genomes. Tumors were only considered integrated if multiple discordant read pairs mapped to similar areas of the human and viral genomes [38]. The ratio of expression of viral genes *E6* and *E7* to *E1* and *E2* has been used as a surrogate marker for integration [39], however, in our hands the ratio of *E6/E7* to *E2/E5* was more correlated to integration identified by discordant read pairs (Figure 3C). Comparison of RNA-based NF-κB activity (classifier scores) demonstrated a strong relationship to viral integration status, with episomal tumors having much higher median NF-κB activity (Figure 3D, *p*-value < 0.001).

### NF-κB activity correlates with patient outcome in an independent validation dataset

**Figure 4:**
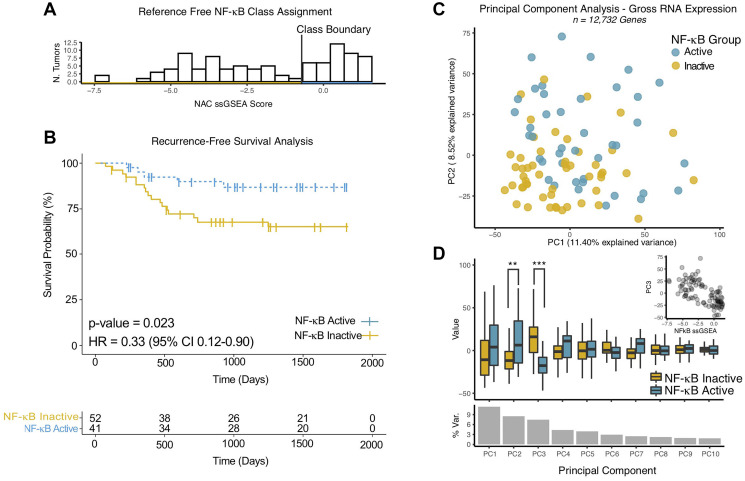
NF-κB activity classifier gene expression is cohesive and correlates with patient outcomes in an independent validation cohort. (**A**) Histogram of single- sample (ss)GSEA Scores for NF-κB activity classifier genes for each tumor in the validation cohort. Class Boundary – an empiric threshold based on the bimodal distribution of scores to assign (binary) NF-κB activity status. (**B**) Kaplan-Meier Analysis of Recurrence Free Survival of HPV+ HNSCC. *P*-values represent log-rank test. HR – Hazard Ratio. NF-κB Active/Inactive – NF-κB active tumors by RNA expression as defined according to the ssGSEA scores for NF-κB activity classifier genes determined for each tumor as in panel A. (**C**) Scatter plot of tumors based on gross RNA expression in principle component space, the top two principal components are displayed. Colors - NF-κB activity groups as in panel A. (**D**) Box Plot of principle component values comparing NF-κB activity groups. *P*-values represent Wilcoxon Rank-sum test. ^**^ *p*-value < 0.001, ^***^ *p*-value < 5 × 10^−9^. % Var. – Percentage of total variance explained by the individual principal component. Inset – Scatter plot of NFkB ssGSEA scores vs. PC3.

To validate the prognostic value of the NAC, we queried the literature for suitable datasets, finding one study with RNAseq data and clinical annotation (Supplementary Table 4, [40]). Since somatic mutational data was not available in this RNA expression dataset, we applied single-sample gene set enrichment analysis (ssGSEA) to score each tumor for NF-κB activity using the NAC (Figure 4A). Interestingly, NAC gene signature ssGSEA scores were distributed in a bimodal pattern, enabling empiric classification of tumors based on a simple threshold roughly dividing the two distributions (Figure 4A). Recurrence-free survival (RFS) analysis demonstrated improved survival for the NF-κB active group (Figure 4B).

### NF-κB activity classifier RNA signature maintains favorable properties in an independent validation dataset

To investigate the relationship to of the NF-κB activity gene signature to global variability in (human) gene expression, we performed principal component (PC) analysis (Figure 4C–4D). NF-κB activity groups were not strongly correlated with the principal component associated with the greatest degree of variability in the dataset (PC1). Among the 10 top principal components, only PC3 (and to a lesser degree PC2), were associated with the NF-κB activity groups (Figure 4C, 4D). Taken together, these results suggest that variability in the expression of the NF-κB activity gene signature is specific, and not simply a reflection of gross data variability. Principal component (PC3) and NAC gene signature ssGSEA scores were strongly correlated (Figure 4D inset, Pearson’s Rho = −0.63, *p*-value = 5 × 10^−12^), which suggests that expression of NF-κB activity signature genes can be reliably identified independent of scoring metric, which is a key feature of high-quality gene signatures [36].

### CYLD missense mutants are not associated with loss of function

**Figure 5:**
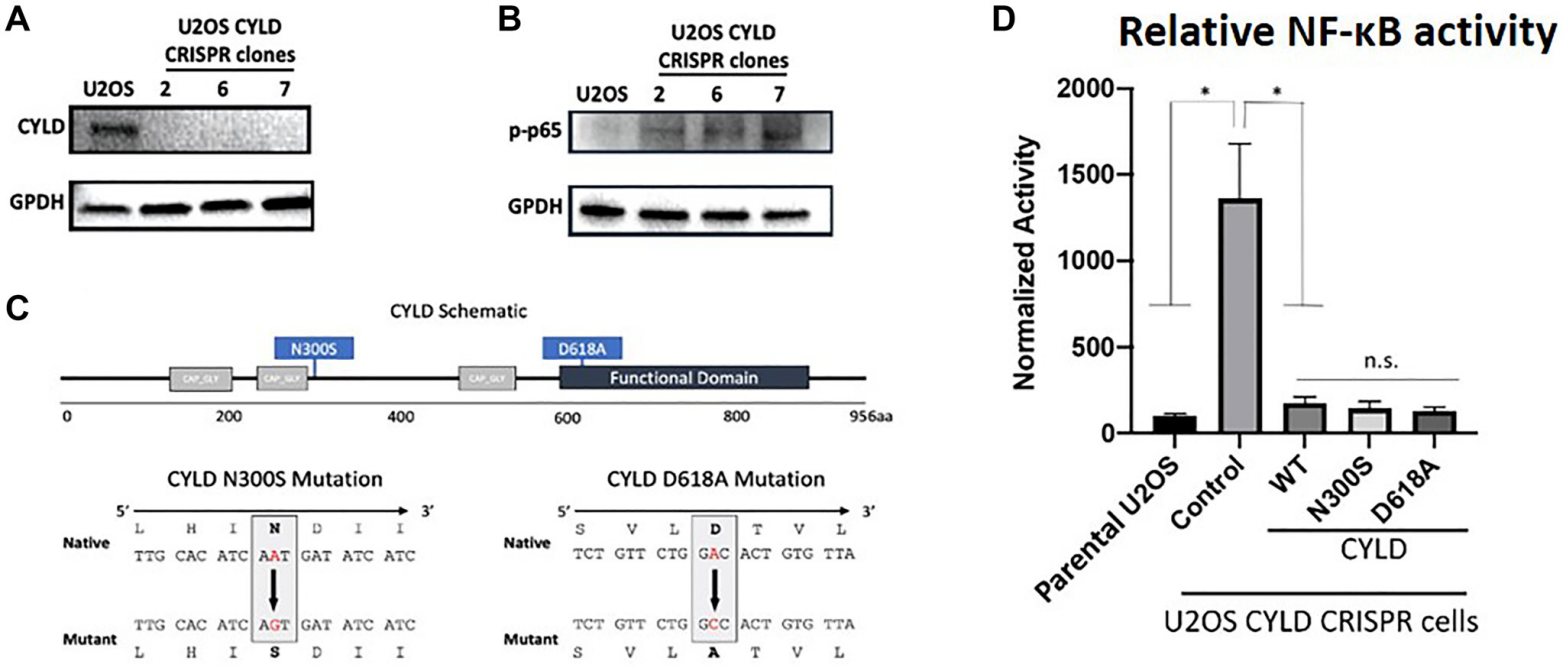
Expression of CYLD (**A**), pp65 (**B**) and GPDH in U2OS parental and CYLD CRISPR clones as determined by immunoblotting. (**C**) Schematic representations of CYLD protein and schema of CYLD N300S and D618A mutant constructions. (**D**) NF-κB reporter activity in U2OS parental, U2OS CYLD CRISPR (control) cells, or U2OS CYLD CRISPR cells transiently transfected with wild-type or mutant CYLD constructs. *t*-test was used to compare U2OS to other conditions. ^**^ -- adjusted *p*-value (Bonferroni correction) < 0.05.

Stratification of tumors by the NF-κB Activity Classifier found that only one of the two identified CYLD missense mutations was associated with increased NF-κB activity (Figure 1, track 8 – lime green). Considering the missense mutation in the “highly active” NF-κB group had concurrent shallow deletions in both *TRAF3* and *CYLD*, we evaluated the functional consequences of the *CYLD* missense mutations. To test CYLD activity, we developed *CYLD* knockout cells and confirmed loss of CYLD expression and activation of NF-κB (Figure 5A, 5B). Site-directed mutagenesis was used to recreate observed mutations (Figure 5C) and activity of mutant proteins to inhibit an NF-κB reporter was compared to wild-type CYLD in CYLD knockout cells. As expected, CYLD knockout cells showed significantly elevated NF-κB activity compared to parental cells (Figure 5D). Interestingly, both N300S and D618A mutant CYLD proteins were as efficient in inhibiting NF-κB transcriptional activity as wild-type CYLD (Figure 5D). These data suggest that N300S and D618A CYLD missense mutations are not inactivating mutations and are not responsible for NF-κB activation.

## DISCUSSION

HNSCC is increasing global incidence due to human papillomavirus and continued consumption of carcinogens [2, 7, 10]. In contrast to HPV-negative HNSCC, HPV-mediated tumors are more susceptible to contemporary treatment paradigms, which also leads to improved patient survival [41]. However, HPV+ HNSCC survivors are frequently burdened with significant side effects including pain; neck muscle stiffness; dry mouth; and difficulty with speech, eating/drinking, and breathing. Efforts to reduce these significant quality-of-life effects have triggered multiple trials of treatment de-escalation. In these trials, patients are selected for deintensified treatment based on patient factors like smoking status, histological characteristics following an ablative procedure, or response to induction chemotherapy [42]. Given that methods to identify patients for deintensified therapy are imperfect, our improved classifiers may serve as prognostic biomarker to help clinicians with therapeutic decisions.

Recent work examined genomic characteristics of the tumor that could be used prior to treatment to prognostically stratify patients. Somatic mutations or deletions in *TRAF3* or *CYLD* identified a subset of HPV+ HNSCC associated with improved outcome [1, 16, 17]. Increasing evidence demonstrates these somatic mutant tumors identify a distinct clinical entity given notable molecular, histopathologic, and outcome differences [3, 16, 43]. Regarding function, TRAF3 is a ubiquitin ligase that regulates numerous receptor pathways, ultimately functioning to negatively regulate both canonical and non-canonical NF-κB pathways [44]. Similarly, CYLD inhibits the NF-κB pathway in its role as a deubiquitinase [45]. Inactivation of TRAF3 or CYLD results in activation of NF-κB producing robust downstream effects as demonstrated by significant RNA expression changes amongst mutant TRAF3/CYLD tumors (Figure 1) [46].

NF-κB was thought to protect cells from viruses through induction of immune response genes; however, it is now apparent that many viruses rely on or even induce aberrant NF-κB activity to promote host cell survival and proliferation, supporting viral lifecycle and gene expression. Previous work revealed that NF-κB overactivation favors carcinogenesis with EBV and HIV-mediated disease with a fundamental role of constitutive NF-κB signaling in EBV tumorigenesis [22, 24–27]. When aberrantly activated, NF-κB stabilizes the EBV episome, while suppressing the lytic cycle [22, 24, 47]. We found that in HPV+ HNSCC TCGA cohort increased NF-κB activity significantly correlated with the absence of HPV integrations (Figure 3C and 3D). Whether constitutively active NF-κB supports the presence of HPV episomes or inhibits HPV integrations in human genome remains to be investigated and currently is studied in our laboratory.

Current knowledge of HPV-induced carcinogenesis is largely derived from study of uterine cervical cancer with the classical model showing persistent infection followed by HPV genome integration leading to increased expression of HPV oncoproteins [48]. The absence of HPV integration in a substantial portion of HNSCC coupled with constitutive NF-κB activation, as we show here (Figure 3), suggests that HPV carcinogenesis in the upper aerodigestive tract may be driven by maintenance of episomal HPV. Interestingly, HPV genome integration has consistently associated with worse survival in these tumors [39, 49, 50].

Recent finding revealed that ER expression correlated with improved survival in HPV+ HNSCC [51]. Initial studies found that ER expression and signaling inhibited NF-κB through estrogen stabilization of IκBα [52]. Later investigations unveiled that ER signaling enhanced NF-κB activity in macrophages and T cells, suggesting that the interaction between ER and NF-κB may depend on cellular context [53, 54]. Given that both ER expression and loss of *TRAF3* portend improved prognosis in HPV+ HNSCC, description that ER-alpha stimulation depletes cells of TRAF3 via ubiquitination provides a potential mechanistic connection of these findings [55]. As far as we are aware, the crosstalk between NF-κB and ER is not described in the presence of HPV and in HNSCC. Although our work cannot determine causality, the WGCNA analysis suggests a positive correlation between ER signaling and NF-κB activity in HPV+ HNSCC, with the “yellow” module being enriched for both NF-κB and early estrogen response genes. Also, the nearest neighbor (relative to “yellow”) “magenta” module was enriched for estrogen response genes (Figure 2A and 2C).

Use of multi-variable predictor models is gaining recent clinical traction since these tools provide a more comprehensive assessment of the intratumoral environment [28–30]. In our case, we hypothesized that undefined alterations in addition to TRAF3 or CYLD gene defects are in play to activate NF-κB in HPV+ HNSCC. Querying only *TRAF3* or *CYLD* defects would be blind to these alternative NF-κB activating strategies leading to imperfect tumor classification. Indeed, the NF-κB Activity Classifier identified several NF-κB active tumors excluded by genomic analysis of *TRAF3/CYLD* (Figure 1A). Tumors with deep deletions in either *TRAF3* or *CYLD*, or a truncating mutation proximal to the proteins’ functional domain, were consistently included in the “active” NF-κB category. Conversely, tumors with isolated shallow deletions tended to be in the NF-κB “inactive” category. However, the NF-κB Activity Classifier identified many samples in the NF-κB “active” category that do not follow this clear-cut pattern, in particular identifying that simultaneous shallow deletion of *TRAF3* and *CYLD* in a tumor correlated with NF-κB activity. The finding that all tumors with shallow co-occurring deletions in both *TRAF3* and *CYLD* were included in the NF-κB “active” group suggests a functional interaction of *TRAF3* and *CYLD* in these tumors. We interrogated tumors without inactivating alterations in TRAF3/CYLD in the NF-κB active group for mutations of genes known to influence the NF-κB pathway; indeed, one tumor contained missense mutation in the *MAP3K14* (NIK), and there was a nonsense mutation in the *NFκBIA*, as well as a nonsense mutation in *TRAF2* in two additional tumors (Supplementary Table 5). However, we were unable to detect additional mutations in well known NF-κB regulators in the rest of tumors most likely due to the complex nature of the NF-κB pathway. On the other hand, our direct testing revealed that missense mutations of *CYLD* found in HPV+ HNSCC do not lose ability to regulate NF-κB (Figure 5). One tumor with the D618A *CYLD* mutation was classified as NF-κB highly active, but this tumor also harbored simultaneous shallow *TRAF3* and *CYLD* deletions. Accuracy of the NF-κB Activity Classifier to identify NF-κB activity in HPV+ HNSCC was suggested through its improved correlation with patient outcome compared to segregating tumors based on *TRAF3* or *CYLD* defects. From the biological perspective, this finding also supports the notion that NF-κB activation and related changes in gene expression may be the key factor determining the biological differences previously reported for *TRAF3/CYLD* mutant HPV+ HNSCC.

Our previous work identified the potential value of *TRAF3* and *CYLD* gene defects to predict outcomes in HPV+ HNSCC [16]. Herein, we demonstrate that an RNA-based classifier trained on tumors harboring these mutations may improve prognostic classification (Figure 3A, 3B, Figure 4B and Supplementary Figure 1). As clinical algorithms for treatment de-escalation are not presently informed by prognostic biomarkers, the possibility of an RNA-based approach for determining NF-κB related prognostic groups is quite relevant. Furthermore, RNA-based gene expression profiling has the potential to synthesize disparate observations related to prognosis in HPV+ OPSCC. Specifically, other groups have found that ER-alpha expression is prognostic [56] and we find that ER signaling is correlated with NF-κB activity (Figure 2C). Similarly, we find that NF-κB activity assessed by RNA expression is highly related to viral integration status which has also been put forward as a prognostic marker in HPV+ OPSCC [39]. Future work will be needed to optimize RNA-based biomarkers which represent the full prognostic potential of all relevant pathways, including NF-κB signaling, ER signaling, and viral oncogene expression, but such a synthetic approach is likely possible based on the correlations between these transcriptional pathways we have identified.

Although application of gene expression sets from translational and experimental studies has only limited success to date, our analyses support the biological and clinical utility of the gene set we have developed. The NF-κB related gene signature and classifier developed in this work demonstrate desirable properties suggesting that they are translatable across multiple cohorts and RNA quantification technologies. Using TCGA data set, we confirmed the robustness of RNA-based classifications in the presence of high levels of noise (Figure 1D). The NF-κB RNA gene set was highly auto-correlated and distinct from other transcriptional programs in HPV+ HNSCC (Figure 1C, Figure 2). Using a second cohort, we validated the utility of our gene set outside of the original training data (Figure 4). In the validation cohort, a bimodal expression of the NF-κB gene signature (Figure 4A) suggests that indeed two biological groups (NF-κB high and low) are a feature of HPV+ HNSCC, and these groups also correlated with RFS in second data set (Figure 4B). Furthermore, the NF-κB gene signature expression was not correlated to 8/10 top principal components demonstrating that the gene set does not simply report gross (transcriptome wide) changes in gene expression. Conversely, the very strong correlation to PC3 suggests that gene set remains compact when applied to new data sets and can likely be quantified by many metrics (Figure 4C, 4D).

This report validates and expands on our findings that significant expression changes related to NF-κB activity occur in the subset of HPV+ HNSCC tumors marked by *TRAF3* or *CYLD* mutations. We are planning future studies investigating the importance of “long-tail” mutations in the NF-κB pathway which might further illuminate the origins of NF-κB dysregulation in HPV+ HNSCC.

A major discovery in the recent past is that HPV associated HNSCC have improved survival compared to tobacco associated tumors. This finding, coupled with advancements in tumor genomic analysis, definitively established HPV+ and HPV-negative HNSCC as distinct tumors. Similarly, we noted genomic differences amongst subclasses of HPV+ HNSCC and found that defects in *TRAF3* and *CYLD* correlated with survival. Here we present data that these subclasses may also be identified by direct assessment of NF-κB activity; as demonstrated by gene expression differences highlighted by the NF-κB Activity Classifier. Since clinicians are exploring therapeutic deintensification for HPV+ HNSCC, identifying patients with good or poor prognosis using the NF-κB Activity Classifier may be useful to guide therapeutic decisions.

## MATERIALS AND METHODS

### Data acquisition

Only de-identified, publicly available clinical and genomic data were utilized for this study. Per-gene quantified mRNA read count data, as well as per-gene discretized Gistic2 copy-number analysis data for the Cancer Genome Atlas [57] HNSCC, were downloaded from the Broad Firehose Portal [58]. In this work, we consider a Gistic score of −2 synonymous with deep deletion, and Gistic score of −1 synonymous with a shallow deletion. Gistic uses a dynamic segmentation algorithm to define chromosomal arm level (−1) and deeper focal deletions (−2) based on per tumor thresholds [59]. Clinical data for the TCGA HNSCC cohort were acquired from Liu et al., [37]. Variant calls were downloaded using the R TCGAbiolinks [60] package; calls performed with VarScan [61] were used for all analyses. TCGA RNA sequencing BAM files were downloaded from dbGaP, with NIH request #99293-1 for project #27853: “Prognostic signature in head and neck cancer” (PI – N.I.).

### Cohort selection and inclusion criteria

RNA assigned HPV status from the Firehose clinical annotations were used to assign HPV status, only HPV positive tumors were included [62]. Tumors with TP53 mutations or deep deletions were excluded from the analysis. Anatomic subsites from the oropharynx, tonsil, and base of tongue were included, and nearby subsites of the hypopharynx and oral tongue considering HPV+ TP53 wild-type tumors were likely an oropharyngeal primary. Tumors from more distal sites (e.g., larynx, alveolar ridge, maxilla) were excluded. A total of 61 patients met these criteria.

### Bioinformatics

RNA read count data was preprocessed by filtering low expression genes to obtain an approximately Gaussian distribution of Log_2_CPM values. Filtered read count data were then normalized using the trimmed means of M values methods provided in the R edgeR package [63]. The Limma-voom pipeline was used for all subsequent differential expression analysis [64]. Classifiers used the nearest centroid method, and were defined and cross validated using the R cancerclass package [65].

To construct a high-performance RNA-based classifier for NF-κB activity in HPV+ HNSCC, we employed a centroid classifier, trained on high confidence class members. Preliminary groups of NF-κB active and inactive tumors were assigned by mutational status. Specifically, all tumors with deep deletions (Gistic value = −2) or mutations (missense, nonsense, frame shift) in the NF-κB regulator genes TRAF3 and CYLD were considered NF-κB active, and other tumors inactive. An initial differential expression was performed between these preliminary groups, and a classifier defined, based on the top 100 genes ranked by *p*-value. High confidence class members were defined as having correct initial assignment and having RNA expression values very similar to the class-defining average of expression (less than 0.25% of the inter-centroid distance). The gene set and classifications were then improved with a machine learning (filtering) procedure, in which tumors initially misclassified or were more than 0.25% away from a centroid were temporarily removed (filtered). Then the filtered data were then used for differential expression and construction of a final classifier. The top 50 genes (by *p*-value) were selected for this final classifier based on lack of improvement in the receiver operator characteristic with the addition of more genes. Adjusted *p*-values (multiple comparison correction per the LIMMA package) were calculated and reported. This final classifier had perfect performance on leave-one-out-cross validation. All tumors in the HPV+ HNSCC cohort were then classified according to this final classifier (nearest centroid method) for correlation with clinical and genomic data. Sample classifications were further tuned by setting an empiric threshold for NF-κB activity at the distance of the frameshift or nonsense TRAF3/CYLD mutation farthest from the NF-κB active centroid.

To identify potentially biologically relevant autocorrelated gene sets or gene expression modules [36], the WGCNA algorithm was applied to the above-described RNA expression data, filtered to the top ~13,000 genes to limit computational intensity. (WGCNA: an R package for weighted correlation network analysis [66]. Default parameters according to recommendations from the WGCNA package authors were used unless otherwise noted. The soft threshold network was constructed calculating a scale-free topology fit index for powers ranging from 4–20. The final scale-free network was constructed with soft power set to 6.

Raw RNAseq reads were analyzed for evidence of viral integration using the ViFi package [38]. Viral genes expression was also quantified using Salmon [67] and the HPV16 A1 genotype, RefSeq NC_001526.4.

### Survival analysis

Clinical data, specifically progression-free interval (PFI), were extracted from Liu et. al. across the full cohort (*n* = 61) [37]. We note that the values for PFI from Liu et al., were very similar or identical (but included four more cases) when compared to recurrence-free survival (RFS) data available from Broad Firehose Portal [58]. Survival statistics were generated with the R survival package (v3.2-7) and visualized with the R survminer package (0.4.8). *p*-values represent log-rank test.

### Gene set enrichment analysis

Ranked gene lists were created using the signal to noise ratio for the change in expression between two groups of interest as defined in the popular GSEA software package distributed by the Broad Institute [68, 69]. Hallmark signatures from the MiSigDB were used as gene sets of interest [70]. GSEA testing and related multiple comparison testing were performed with the R fgsea package [71]. Hypergeometric (gene ontology) enrichment analysis was performed for the derived WGCNA modules using the EnrichR package with default parameters [72]. All results were corrected for multiple comparisons by the EnrichR pipeline, and adjusted *p*-values were considered significant if adjusted *p* < 0.05.

### Evaluating the TCGA mutational landscape

The *TRAF3/CYLD* mutational loci and type were assessed across HPV+ HNSCC tumors. *TRAF3* genetic alterations were predominantly deep deletions as well as two truncations; these alterations preclude translation of the TRAF3 ubiquitin ligase enzymatic domain resulting in this NF-κB overactive phenotype. Similarly, *CYLD* alterations included deep deletions and truncations occurring prior to its de-ubiquitinase functional domain [1]. In both cases, protein loss of function is evident, leading to unchecked NF-κB activation. However, two novel *CYLD* missense mutations (N300S and D618A) with unknown functional significance were discovered, demanding further functional appraisal.

### Modeling the novel CYLD missense mutations

Employing the QuikChange II-E Site-Directed Mutagenesis Kit (Agilent #200523) per the manufacture’s protocol, a wild-type Flag-HA-CYLD expression vector [73]. (Addgene #22544) was mutated to reflect the two novel *CYLD* missense mutations, N300S and D618A. Synthetic forward and reverse oligonucleotide primers (Sigma-Aldrich) were designed to harbor the desired point mutation with high CYLD binding affinity in the region of interest. To create the N300S *CYLD* mutation, forward primer ACATCAGTGATATCATCCCAGCTTTAT and reverse primer GCAATAGAATTGTACTTTCAACACACG were used. To develop the D618A CYLD mutation, gggtctaagtaacacagtggccagaacagaactaaaagc and gcttttagttctgttctggccactgtgttacttagaccc were used for the forward and reverse primers, respectively. Sanger sequencing performed by Eton Bioscience (San Diego, CA, USA) confirmed targeted mutation success.

### Creation of CYLD knockout mammalian cells

Co-transfection of CYLD CRISPR/Cas KO (Santa Cruz #sc-400882-KO-2) and CYLD HDR (Santa Cruz #sc-400882-HDR-2) plasmids were used per manufacture’s protocol to develop CYLD knockout U2OS cells. U2OS was chosen as the parental cell based on known wild-type *TP53* and *Rb* expression, characteristic of HPV+ HNSCC disease [74]. Cells were grown in 5% CO_2_ at 37^°^C in DMEM (Genesee #25-501N) supplemented with 10% FBS (Genesee #25-514H) and 1% each of penicillin-streptomycin (Genesee #25-512), non-essential amino acids (Genesee #25-536), and glutamine (Genesee #25-509). KO CYLD cell media was further supplemented with 1 μg/ml puromycin (InvivoGen ant-pr-1) used to select for CRISPR-Cas9 clones. Confirmation of CYLD knockdown was performed with Western blot and a luciferase NF-κB functional assay.

### Western blot

Cells were collected by trypsinization and lysed in radioimmunoprecipitation assay (RIPA lysis buffer (Sigma) with the addition of protease inhibitors (Roche) and phosphatase inhibitors (Sigma) for 15 minutes on ice. Lysates were then mechanically homogenized with an 18-gauge syringe and insoluble material was removed by centrifugation at 14,000 rpm for 15 minutes at 4^°^C. Protein concentration was determined using Qubit assay (Invitrogen). Twenty micrograms of total protein were mixed with 2X loading Laemmli buffer (Biorad) supplemented with DTT (Sigma) and incubated for 10 minutes at 95^°^C. Proteins were separated in 4% to 20% Tris-glycine polyacrylamide gels (Mini-PROTEAN; Bio-Rad) and electrophoretically transferred onto polyvinylidene fluoride membranes. Membranes were blocked with 3% BSA in PBS and incubated with primary antibodies against CYLD (Santa Cruz) and phospho-p65 (Cell Signaling) as well as control primary antibodies against GAPDH (Santa Cruz). Secondary antibodies were conjugated with horseradish peroxidase (Cell Signaling). After sequential washes in TBST buffer, a chemiluminescent HRP substrate was applied to the membrane and signals were immediately visualized using a ChemiDoc Bio-Rad imager.

### *In vitro* NF-κB functional evaluation

U2OS and U2OS CYLD KO cells were plated in a 96 well plate at 5 × 10^4^ cells/100 μl/well. After 24 hours, cells were co-transfected with a 3κB-conA-luciferase expression vector (a generous gift from Dr. Neil Perkins of the University of Dundee, Dundee, UK) and either a CYLD wild-type, CYLD N300S, CYLD D618A, or an empty expression vector using a lipofectamine 2000 (Thermo Fisher #11668030) system per manufacturer’s protocol. Forty-eight hours following transfection, cells were lysed and luciferin was applied per manufacturer’s protocol (Promega #E1501). Luciferase activity was measured using Promega GloMax Explorer.

## SUPPLEMENTARY MATERIALS









## Abbreviations

HNSCC: head and neck squamous cell carcinoma
HPV: human papilloma virus
HPV+: human papilloma virus associated
NAC: NF-κB Activity Classifier
NPC: nasopharyngeal carcinoma
ML: Machine Learning
AUC: receiver-operator characteristic curve
WGCNA: weighted gene correlation network analysis
ssGSEA: single-sample gene set enrichment analysis
ER: estrogen receptor
PC: principal component
PCA: principal component analysis
OPSCC: oropharyngeal squamous cell carcinoma
