# Supplementary material for: NF-κB over-activation portends improved outcomes in HPV-associated head and neck cancer
Source: Oncotarget. 2022 May 24;13:707–22. doi: 10.18632/oncotarget.28232 (PMC9131933; doi:10.18632/oncotarget.28232)
Supplement: Supplementary file 1 [file oncotarget-13-28232-s001.pdf]

NF-κB over-activation portends improved outcomes in HPV-associated head and neck cancer

SUPPLEMENTARY MATERIALS

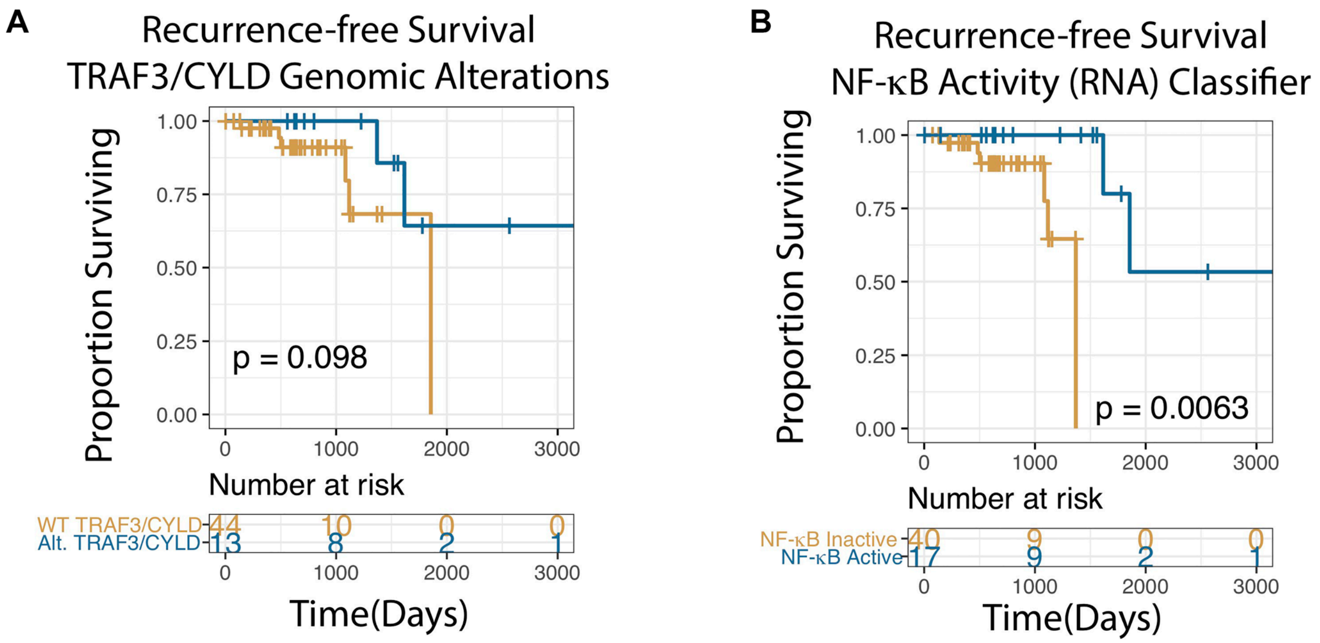

Supplementary Figure 1: Kaplan-Meier analysis of recurrence free survival of HPV+ HNSCC. *P*-values represent log-rank test. (**A**) Survival of patients with and without TRAF3/CYLD altered tumors. Alteration defined as any missense, nonsense, frameshift, deep deletion in TRAF3 and/or CYLD, these were compared to all other tumors (TRAF3/CYLD WT) in the study cohort. (**B**) Survival of patients with and without highly NF-κB active (NF-κB Inactive) tumors by RNA expression as defined according to the RNA based classifier (see methods), these were compared to all other tumors (NF-κB Inactive) in the study cohort. See Figure 3 for grossly similar progression-free survival results.

Supplementary Table 1: Genes in the final NF-kB classifier

HUGO Gene Name	Log Fold-Change	Adjusted *P*-Value
MGAT3	4.72834177	5.23E-13
STAR	4.345735136	1.14E-11
VCAM1	4.679985591	6.61E-11
RAB42	3.163067177	2.29E-10
NFE2L3	2.307053108	9.15E-10
FGF2	3.119125796	9.15E-10
ABCA3	4.725320799	9.15E-10
RNF165	2.887336939	1.76E-09
PKDCC	4.968886543	1.85E-09
ZBTB46	2.079653042	2.89E-09
IL27RA	2.812122457	3.31E-09
KREMEN2	4.260022489	3.81E-09
ARNT2	3.676622025	8.49E-09
MMP19	2.00769653	1.57E-08
PARM1	3.827746878	2.17E-08
VRK2	1.430805242	2.42E-08
COL22A1	4.814102899	2.42E-08
BIRC3	2.851140525	3.60E-08
SIM2	3.372941806	4.61E-08
MEGF10	4.809881389	5.99E-08
MAP3K14	1.840334773	7.04E-08
C9orf172	2.958009915	5.68E-08
C11orf92	5.409698384	7.04E-08
CDH23	3.621303931	7.04E-08
C8orf42	3.107301157	9.46E-08
ERO1LB	1.982118254	9.46E-08
TMEM150C	2.808088143	9.46E-08
SV2B	4.246695942	9.31E-08
FAM105B	1.075846134	1.24E-07
C9orf98	3.28370688	1.05E-07
CYP27A1	3.40525234	1.27E-07
LIFR	3.050401304	1.30E-07
RTN4RL1	3.925200083	1.86E-07
LOC283174	3.619050677	1.80E-07
MCF2L	2.171658374	2.62E-07
NEDD1	1.325230936	2.62E-07
LOC100272146	1.447442118	2.20E-07
TLR6	2.926082298	2.58E-07
GALNT11	1.420574567	4.66E-07
CDRT4	1.347258909	5.23E-07
NT5DC1	1.230726852	5.23E-07
TRAF2	1.851754942	5.98E-07
FAM65C	3.195180329	6.01E-07
ITGAM	2.671205127	6.72E-07

ZNF488	2.375328198	7.30E-07
RELB	1.919392442	7.30E-07
VSTM2L	4.198787459	7.72E-07
LGI2	4.186955961	8.37E-07
FAM164A	1.861510971	8.55E-07
NOXO1	3.164931786	7.72E-07

Log Fold-Change and Adjusted *P*-Values were generated with LIMMA, comparing differential expression of classifier genes when comparing of true-positives and true-negatives cases based on the initial (unimproved) classifier, see Methods.

Supplementary Table 2: Sets of highly autocorrelated genes after weighted gene correlation network analysis (WGCNA). See Supplementary Table 2

Supplementary Table 3: Hypergeometric enrichment analysis comparing WGCNA modules and MISigDB hallmark gene sets. See Supplementary Table 3

Supplementary Table 4: Clinical characteristics of vanderbilt cohort of HPV+ HNSCC patients

		NFkB Inactive	NFkB Active	*p*-value
		***n* = 52**	***n* = 41**	
Pathologic N Stage (%)	N0	3 (13.0)	3 (15.8)	0.31
	N1	7 (30.4)	2 (10.5)	
	N2	12 (52.2)	14 (73.7)	
	N3	1 (4.3)	0 (0.0)	
Pathologic T Stage (%)	T0	1 (4.3)	2 (9.5)	0.152
	T1	13 (56.5)	14 (66.7)	
	T2	9 (39.1)	3 (14.3)	
	T3	0 (0.0)	2 (9.5)	
Pathologic Summary Stage (%)	Stage 1	1 (5.3)	0 (0.0)	0.773
	Stage 2	2 (10.5)	1 (6.7)	
	Stage 3	3 (15.8)	2 (13.3)	
	Stage 4	13 (68.4)	12 (80.0)	
Treatment Strategy (%)	S	6 (12.0)	3 (7.5)	0.334
	S+CXRT	21 (42.0)	23 (57.5)	
	CXRT	23 (46.0)	14 (35.0)	
Race (%)	Other	0 (0.0)	2 (4.9)	0.373
	White	52 (100.0)	39 (95.1)	
Sex (%)	F	2 (3.8)	5 (12.2)	0.263
	M	50 (96.2)	36 (87.8)	
Smoking (%)	Never Smoker	17 (32.7)	17 (42.5)	0.454
	Smoker	35 (67.3)	23 (57.5)	
Age (%)	<50	16 (30.8)	8 (19.5)	0.321
	≥50	36 (69.2)	33 (80.5)	

Abbreviations: XRT: Radiation Therapy; CXRT: Chemoradiation Therapy; S: Surgery.

Supplementary Table 5: Alterations in the NF-κB pathway found in the HPV+ TCGA cohort. See Supplementary Table 5.
