# Supplementary material for: NF-κB over-activation portends improved outcomes in HPV-associated head and neck cancer
Source: Oncotarget. 2022 May 24;13:707–22. doi: 10.18632/oncotarget.28232 (PMC9131933; doi:10.18632/oncotarget.28232)
Supplement: Supplementary file 2 [file oncotarget-13-28232-s002.docx]

**Supplementary Table 2: Sets of highly autocorrelated genes after weighted gene correlation network analysis (WGCNA)**

| WGCNA Module Name | Hugo Gene Name |
| --- | --- |
| blue | A2LD1 |
| magenta | A2ML1 |
| brown | A2M |
| yellow | AACS |
| yellow | ABCA17P |
| yellow | ABCA3 |
| blue | ABCA7 |
| yellow | ABCC4 |
| brown | ABCC9 |
| pink | ABCD1 |
| grey | ABCF2 |
| brown | ABCG1 |
| brown | ABHD3 |
| grey | ABHD4 |
| magenta | ABI2 |
| blue | ABI3BP |
| blue | ABI3 |
| grey | ABLIM3 |
| brown | ABP1 |
| yellow | ABTB2 |
| brown | ACAA2 |
| grey | ACACB |
| red | ACAD11 |
| blue | ACAP1 |
| brown | ACAT2 |
| brown | ACBD7 |
| blue | ACCN2 |
| green | ACD |
| pink | ACE |
| brown | ACIN1 |
| green | ACO2 |
| magenta | ACOT11 |
| pink | ACP2 |
| pink | ACP5 |
| brown | ACSL1 |
| brown | ACTA2 |
| pink | ACTB |
| blue | ACTG1 |
| brown | ACTG2 |
| brown | ACTN1 |
| brown | ACTR6 |
| red | ACVR1 |
| blue | ACVR2A |
| brown | ACVRL1 |
| red | ACYP1 |
| grey | ADAL |
| brown | ADAM12 |
| magenta | ADAM15 |
| yellow | ADAM19 |
| brown | ADAM23 |
| blue | ADAM28 |
| blue | ADAM6 |
| yellow | ADAM8 |
| blue | ADAMDEC1 |
| brown | ADAMTS12 |
| brown | ADAMTS14 |
| yellow | ADAMTS17 |
| brown | ADAMTS2 |
| brown | ADAMTS4 |
| brown | ADAMTS7 |
| brown | ADAMTS9 |
| brown | ADAMTSL2 |
| blue | ADAMTSL5 |
| pink | ADAP2 |
| blue | ADARB1 |
| yellow | ADARB2 |
| red | ADAT2 |
| green | ADAT3 |
| green | ADCK2 |
| brown | ADCY1 |
| brown | ADCY4 |
| brown | ADCY5 |
| green | ADCY6 |
| yellow | ADC |
| grey | ADH5 |
| grey | ADH7 |
| blue | ADM |
| blue | ADORA2A |
| grey | ADORA2B |
| pink | ADORA3 |
| grey | ADO |
| blue | ADPGK |
| blue | ADPRH |
| blue | ADRA2A |
| brown | ADRB2 |
| blue | ADRBK2 |
| brown | AEBP1 |
| green | AEN |
| green | AES |
| green | AFAP1L1 |
| yellow | AFAP1L2 |
| red | AFG3L1 |
| grey | AFG3L2 |
| brown | AG2 |
| blue | AGAP2 |
| red | AGAP4 |
| red | AGAP6 |
| green | AGA |
| blue | AGBL5 |
| red | AGER |
| grey | AGMAT |
| yellow | AGPAT3 |
| magenta | AGPAT4 |
| grey | AGR2 |
| red | AHSA2 |
| grey | AIF1L |
| pink | AIF1 |
| blue | AIG1 |
| magenta | AIM1L |
| yellow | AK3L1 |
| brown | AKAP12 |
| blue | AKAP5 |
| blue | AKAP7 |
| brown | AKAP8 |
| grey | AKIRIN2 |
| blue | AKNA |
| magenta | AKR1B10 |
| yellow | AKR1C1 |
| yellow | AKR1C2 |
| yellow | AKR1C3 |
| grey | AKT2 |
| grey | ALDH1A1 |
| brown | ALDH1B1 |
| brown | ALDH1L2 |
| yellow | ALDH2 |
| grey | ALDH3A1 |
| grey | ALDH3A2 |
| pink | ALDH3B1 |
| magenta | ALDH3B2 |
| grey | ALDH4A1 |
| blue | ALDH5A1 |
| brown | ALDH7A1 |
| blue | ALDH9A1 |
| green | ALDOA |
| green | ALG1 |
| grey | ALG2 |
| brown | ALG6 |
| grey | ALG8 |
| brown | ALKBH1 |
| green | ALKBH2 |
| grey | ALKBH5 |
| green | ALKBH7 |
| blue | ALOX12 |
| pink | ALOX15B |
| pink | ALOX5AP |
| pink | ALOX5 |
| green | ALPK1 |
| yellow | ALPK2 |
| brown | ALPL |
| pink | ALS2CR4 |
| grey | ALX3 |
| blue | AMACR |
| blue | AMICA1 |
| brown | AMIGO2 |
| grey | AMN1 |
| brown | AMOT |
| brown | AMPD2 |
| yellow | AMPD3 |
| yellow | AMTN |
| red | AMT |
| red | AMY2B |
| green | AMZ2 |
| green | ANAPC7 |
| brown | ANGPT2 |
| brown | ANGPTL2 |
| grey | ANGPTL4 |
| green | ANK1 |
| brown | ANK2 |
| blue | ANKDD1A |
| yellow | ANKH |
| yellow | ANKLE2 |
| red | ANKMY1 |
| pink | ANKMY2 |
| magenta | ANKRD13B |
| green | ANKRD16 |
| yellow | ANKRD29 |
| red | ANKRD36 |
| grey | ANKRD37 |
| magenta | ANKRD56 |
| red | ANKS3 |
| grey | ANKS6 |
| red | ANKZF1 |
| yellow | ANO4 |
| grey | ANO8 |
| brown | ANPEP |
| brown | ANTXR2 |
| magenta | ANXA1 |
| magenta | ANXA2P1 |
| magenta | ANXA2P2 |
| magenta | ANXA2P3 |
| magenta | ANXA2 |
| blue | ANXA3 |
| grey | ANXA4 |
| brown | ANXA5 |
| blue | ANXA6 |
| grey | ANXA8L2 |
| grey | ANXA8 |
| pink | AOAH |
| brown | AOC3 |
| brown | AOX1 |
| red | AP1B1 |
| green | AP1M1 |
| yellow | AP1M2 |
| pink | AP1S2 |
| grey | AP2A1 |
| brown | AP2B1 |
| grey | AP3B2 |
| green | AP3D1 |
| magenta | AP3M2 |
| grey | AP3S2 |
| brown | APBA2 |
| green | APBA3 |
| blue | APBB1IP |
| brown | APBB2 |
| red | APBB3 |
| brown | APCDD1 |
| blue | APH1A |
| pink | APH1B |
| green | APLF |
| brown | APLNR |
| blue | APLN |
| pink | APOB48R |
| brown | APOBEC3B |
| blue | APOBEC3D |
| blue | APOBEC3F |
| blue | APOBEC3G |
| pink | APOC1 |
| pink | APOC2 |
| brown | APOD |
| pink | APOE |
| pink | APOL4 |
| brown | APOLD1 |
| green | APTX |
| brown | AQP1 |
| magenta | AQP3 |
| blue | AQP5 |
| pink | ARAP1 |
| blue | ARAP3 |
| green | ARF3 |
| grey | ARG2 |
| red | ARGLU1 |
| blue | ARHGAP15 |
| pink | ARHGAP18 |
| yellow | ARHGAP22 |
| magenta | ARHGAP23 |
| blue | ARHGAP25 |
| yellow | ARHGAP26 |
| magenta | ARHGAP27 |
| brown | ARHGAP28 |
| blue | ARHGAP30 |
| yellow | ARHGAP31 |
| red | ARHGAP33 |
| blue | ARHGAP9 |
| blue | ARHGDIB |
| magenta | ARHGEF10L |
| brown | ARHGEF15 |
| brown | ARHGEF16 |
| brown | ARHGEF17 |
| green | ARHGEF18 |
| blue | ARHGEF1 |
| brown | ARHGEF2 |
| magenta | ARHGEF37 |
| magenta | ARHGEF4 |
| blue | ARHGEF6 |
| blue | ARID5A |
| brown | ARL4C |
| yellow | ARL4D |
| blue | ARL6IP5 |
| magenta | ARL8B |
| green | ARMC6 |
| brown | ARMC9 |
| brown | ARMCX1 |
| yellow | ARNT2 |
| yellow | ARPC1A |
| yellow | ARRB1 |
| pink | ARRB2 |
| green | ARSB |
| green | ARSD |
| grey | ARSI |
| pink | ASAH1 |
| brown | ASAP3 |
| brown | ASB1 |
| grey | ASB2 |
| red | ASB6 |
| grey | ASB8 |
| yellow | ASB9 |
| grey | ASCC1 |
| grey | ASF1A |
| brown | ASF1B |
| green | ASNA1 |
| grey | ASNSD1 |
| brown | ASPN |
| brown | ASRGL1 |
| brown | ASTE1 |
| yellow | ASTN2 |
| grey | ATAD1 |
| red | ATAD3B |
| yellow | ATF5 |
| blue | ATF7IP2 |
| grey | ATG16L1 |
| red | ATG16L2 |
| grey | ATG2A |
| green | ATG4D |
| grey | ATG5 |
| grey | ATG9A |
| red | ATG9B |
| red | ATHL1 |
| brown | ATL1 |
| yellow | ATOH8 |
| brown | ATP10A |
| magenta | ATP10B |
| green | ATP13A1 |
| yellow | ATP13A2 |
| magenta | ATP13A4 |
| grey | ATP1A1 |
| blue | ATP1B1 |
| yellow | ATP1B3 |
| blue | ATP2A3 |
| yellow | ATP2C2 |
| green | ATP5A1 |
| green | ATP5B |
| green | ATP5D |
| green | ATP5SL |
| yellow | ATP6AP2 |
| blue | ATP6V1B2 |
| blue | ATP8A1 |
| blue | ATP8B2 |
| brown | ATPBD4 |
| blue | ATXN10 |
| red | ATXN7L2 |
| brown | AUH |
| brown | AURKA |
| brown | AURKB |
| green | AXIN1 |
| brown | AXIN2 |
| brown | AXL |
| grey | B3GALTL |
| grey | B3GNT3 |
| magenta | B3GNT7 |
| magenta | B3GNT8 |
| blue | B3GNT9 |
| grey | B4GALNT1 |
| blue | B4GALNT4 |
| brown | B4GALT1 |
| yellow | B4GALT3 |
| yellow | B4GALT6 |
| brown | BACE1 |
| blue | BACE2 |
| yellow | BAI2 |
| blue | BAIAP2L1 |
| blue | BAIAP2 |
| blue | BANK1 |
| grey | BARX1 |
| magenta | BARX2 |
| blue | BASP1 |
| blue | BATF |
| green | BBS12 |
| green | BBS4 |
| grey | BBS5 |
| green | BBS7 |
| green | BBS9 |
| magenta | BCAS1 |
| brown | BCAT1 |
| green | BCKDK |
| magenta | BCL10 |
| blue | BCL11A |
| blue | BCL11B |
| blue | BCL2A1 |
| green | BCL2L12 |
| red | BCL2L13 |
| blue | BCL2L14 |
| grey | BCL2L2 |
| yellow | BCL2 |
| grey | BCL3 |
| brown | BCL6B |
| magenta | BCL7A |
| green | BCL9L |
| blue | BCR |
| yellow | BDH1 |
| magenta | BDKRB2 |
| yellow | BECN1 |
| brown | BEX2 |
| brown | BGN |
| yellow | BHLHE41 |
| blue | BIK |
| blue | BIN2 |
| yellow | BIRC3 |
| blue | BLK |
| blue | BLNK |
| brown | BMF |
| brown | BMP1 |
| yellow | BMP2 |
| brown | BMP6 |
| grey | BMP7 |
| brown | BMP8A |
| blue | BMPR1B |
| grey | BMS1 |
| brown | BNC2 |
| magenta | BNIPL |
| brown | BOC |
| magenta | BPNT1 |
| green | BRMS1L |
| green | BSG |
| blue | BTBD10 |
| magenta | BTBD11 |
| green | BTBD2 |
| grey | BTD |
| yellow | BTF3L4 |
| blue | BTG1 |
| brown | BTG3 |
| blue | BTK |
| yellow | BTNL9 |
| brown | BUB3 |
| green | BVES |
| red | BZRAP1 |
| grey | BZW2 |
| yellow | C10orf10 |
| brown | C10orf137 |
| brown | C10orf26 |
| blue | C10orf54 |
| magenta | C10orf57 |
| yellow | C10orf72 |
| brown | C10orf78 |
| grey | C10orf81 |
| green | C10orf88 |
| magenta | C10orf99 |
| yellow | C11orf41 |
| magenta | C11orf46 |
| grey | C11orf54 |
| brown | C11orf57 |
| yellow | C11orf58 |
| red | C11orf61 |
| green | C11orf84 |
| yellow | C11orf92 |
| yellow | C11orf93 |
| brown | C11orf95 |
| blue | C11orf9 |
| green | C12orf10 |
| yellow | C12orf23 |
| blue | C12orf26 |
| yellow | C12orf34 |
| magenta | C12orf41 |
| brown | C12orf56 |
| green | C12orf5 |
| red | C12orf76 |
| pink | C13orf15 |
| blue | C13orf18 |
| grey | C13orf1 |
| brown | C13orf29 |
| green | C13orf31 |
| brown | C13orf33 |
| magenta | C14orf129 |
| green | C14orf132 |
| blue | C14orf139 |
| yellow | C14orf147 |
| brown | C14orf169 |
| yellow | C14orf73 |
| brown | C15orf23 |
| yellow | C15orf29 |
| magenta | C15orf39 |
| grey | C15orf44 |
| blue | C15orf57 |
| brown | C16orf45 |
| blue | C16orf54 |
| grey | C16orf73 |
| blue | C16orf74 |
| brown | C16orf75 |
| brown | C17orf28 |
| grey | C17orf51 |
| brown | C17orf53 |
| red | C17orf56 |
| grey | C17orf58 |
| red | C17orf65 |
| blue | C17orf68 |
| red | C17orf86 |
| green | C17orf97 |
| grey | C18orf10 |
| yellow | C18orf1 |
| green | C18orf55 |
| green | C18orf8 |
| green | C19orf10 |
| pink | C19orf12 |
| green | C19orf20 |
| blue | C19orf21 |
| green | C19orf22 |
| green | C19orf24 |
| green | C19orf25 |
| green | C19orf28 |
| green | C19orf29 |
| magenta | C19orf33 |
| red | C19orf36 |
| brown | C19orf40 |
| green | C19orf43 |
| red | C19orf44 |
| green | C19orf50 |
| green | C19orf52 |
| green | C19orf53 |
| green | C19orf54 |
| green | C19orf56 |
| green | C19orf57 |
| green | C19orf60 |
| green | C19orf62 |
| green | C19orf6 |
| green | C19orf70 |
| pink | C1QA |
| green | C1QBP |
| pink | C1QB |
| pink | C1QC |
| yellow | C1QTNF1 |
| brown | C1QTNF3 |
| brown | C1QTNF6 |
| green | C1RL |
| brown | C1R |
| brown | C1S |
| magenta | C1orf106 |
| red | C1orf113 |
| magenta | C1orf116 |
| magenta | C1orf126 |
| brown | C1orf131 |
| brown | C1orf135 |
| grey | C1orf144 |
| pink | C1orf162 |
| magenta | C1orf170 |
| brown | C1orf172 |
| brown | C1orf174 |
| red | C1orf175 |
| brown | C1orf198 |
| yellow | C1orf201 |
| magenta | C1orf210 |
| yellow | C1orf21 |
| blue | C1orf226 |
| pink | C1orf38 |
| pink | C1orf54 |
| red | C1orf63 |
| blue | C1orf74 |
| yellow | C1orf93 |
| magenta | C20orf108 |
| yellow | C20orf112 |
| yellow | C20orf54 |
| brown | C21orf45 |
| grey | C21orf56 |
| red | C21orf58 |
| grey | C22orf13 |
| grey | C22orf23 |
| yellow | C22orf28 |
| brown | C22orf46 |
| blue | C2CD2L |
| yellow | C2CD2 |
| green | C2CD4B |
| magenta | C2orf29 |
| blue | C2orf43 |
| magenta | C2orf55 |
| red | C2orf56 |
| grey | C2orf65 |
| green | C2orf67 |
| brown | C2orf77 |
| green | C2orf79 |
| pink | C2 |
| pink | C3AR1 |
| grey | C3orf14 |
| blue | C3orf52 |
| blue | C3orf57 |
| blue | C3orf59 |
| green | C3orf64 |
| blue | C3 |
| brown | C4A |
| yellow | C4orf14 |
| magenta | C4orf19 |
| grey | C4orf33 |
| green | C4orf34 |
| green | C4orf41 |
| grey | C4orf43 |
| blue | C4orf7 |
| pink | C5AR1 |
| brown | C5orf13 |
| brown | C5orf15 |
| blue | C5orf20 |
| grey | C5orf23 |
| red | C5orf34 |
| brown | C5orf35 |
| blue | C5orf39 |
| blue | C5orf53 |
| grey | C5orf54 |
| blue | C5orf56 |
| brown | C5orf62 |
| yellow | C6orf105 |
| magenta | C6orf132 |
| red | C6orf134 |
| yellow | C6orf141 |
| green | C6orf162 |
| yellow | C6orf168 |
| grey | C6orf182 |
| blue | C6orf223 |
| blue | C6orf64 |
| grey | C7orf25 |
| grey | C7orf28B |
| blue | C7orf29 |
| blue | C7orf31 |
| brown | C7orf42 |
| yellow | C7orf44 |
| brown | C7orf46 |
| yellow | C7orf49 |
| brown | C7orf58 |
| blue | C7orf68 |
| grey | C7orf70 |
| blue | C7 |
| green | C8orf38 |
| green | C8orf41 |
| yellow | C8orf42 |
| yellow | C8orf4 |
| magenta | C8orf73 |
| grey | C8orf79 |
| brown | C9orf100 |
| blue | C9orf125 |
| brown | C9orf140 |
| brown | C9orf150 |
| grey | C9orf21 |
| grey | C9orf25 |
| blue | C9orf30 |
| green | C9orf40 |
| red | C9orf45 |
| yellow | C9orf85 |
| blue | C9orf91 |
| yellow | C9orf98 |
| magenta | CA12 |
| yellow | CA2 |
| grey | CA9 |
| brown | CAB39L |
| brown | CABLES2 |
| grey | CACNA1B |
| brown | CACNA1C |
| brown | CACNA1H |
| brown | CADM1 |
| brown | CADM3 |
| blue | CADM4 |
| yellow | CADPS2 |
| yellow | CALB1 |
| brown | CALCRL |
| brown | CALD1 |
| blue | CALHM2 |
| magenta | CALML3 |
| red | CALML4 |
| brown | CALU |
| pink | CAMK1 |
| green | CAMK2D |
| brown | CAMK2N1 |
| red | CANT1 |
| red | CAPN10 |
| magenta | CAPN14 |
| blue | CAPN1 |
| magenta | CAPN2 |
| magenta | CAPN5 |
| red | CAPRIN2 |
| grey | CARD10 |
| blue | CARD11 |
| magenta | CARD14 |
| pink | CARD16 |
| blue | CARD8 |
| blue | CARD9 |
| green | CARM1 |
| grey | CASP3 |
| blue | CASP6 |
| green | CASP8 |
| brown | CASP9 |
| brown | CAT |
| green | CAV1 |
| green | CAV2 |
| blue | CBARA1 |
| brown | CBFA2T3 |
| blue | CBLC |
| yellow | CBLN2 |
| yellow | CBLN3 |
| green | CBR4 |
| brown | CBS |
| brown | CBWD6 |
| blue | CBX1 |
| brown | CBX2 |
| grey | CBX4 |
| yellow | CBX7 |
| green | CC2D1A |
| blue | CC2D2A |
| grey | CCBL2 |
| green | CCDC111 |
| magenta | CCDC120 |
| green | CCDC123 |
| green | CCDC124 |
| brown | CCDC125 |
| red | CCDC130 |
| grey | CCDC134 |
| yellow | CCDC149 |
| red | CCDC150 |
| grey | CCDC25 |
| yellow | CCDC28B |
| yellow | CCDC3 |
| blue | CCDC43 |
| red | CCDC45 |
| red | CCDC57 |
| magenta | CCDC64B |
| brown | CCDC64 |
| green | CCDC68 |
| blue | CCDC69 |
| grey | CCDC77 |
| brown | CCDC80 |
| green | CCDC86 |
| blue | CCDC88B |
| yellow | CCDC8 |
| grey | CCDC90B |
| green | CCDC94 |
| yellow | CCDC97 |
| green | CCDC9 |
| pink | CCL18 |
| blue | CCL19 |
| yellow | CCL20 |
| blue | CCL21 |
| blue | CCL22 |
| pink | CCL2 |
| pink | CCL3 |
| blue | CCL4L2 |
| blue | CCL4 |
| blue | CCL5 |
| brown | CCNB1 |
| brown | CCNB2 |
| yellow | CCND1 |
| blue | CCND2 |
| grey | CCNDBP1 |
| brown | CCNF |
| blue | CCNG1 |
| green | CCNG2 |
| yellow | CCNJL |
| red | CCNL2 |
| pink | CCR1 |
| blue | CCR2 |
| blue | CCR4 |
| blue | CCR5 |
| blue | CCR6 |
| blue | CCR7 |
| green | CCT5 |
| blue | CD101 |
| pink | CD14 |
| pink | CD163 |
| grey | CD177 |
| blue | CD180 |
| blue | CD19 |
| blue | CD1A |
| blue | CD1E |
| yellow | CD200 |
| blue | CD207 |
| pink | CD209 |
| blue | CD22 |
| blue | CD247 |
| brown | CD248 |
| magenta | CD24 |
| blue | CD274 |
| brown | CD276 |
| blue | CD27 |
| blue | CD28 |
| blue | CD2 |
| pink | CD300A |
| pink | CD300LF |
| yellow | CD302 |
| green | CD320 |
| brown | CD34 |
| brown | CD36 |
| blue | CD37 |
| blue | CD38 |
| blue | CD3D |
| blue | CD3E |
| blue | CD3G |
| blue | CD40 |
| brown | CD47 |
| blue | CD48 |
| pink | CD4 |
| blue | CD52 |
| blue | CD53 |
| blue | CD55 |
| yellow | CD59 |
| blue | CD5 |
| pink | CD68 |
| blue | CD69 |
| blue | CD6 |
| blue | CD72 |
| blue | CD74 |
| blue | CD79A |
| blue | CD79B |
| blue | CD7 |
| pink | CD81 |
| grey | CD82 |
| blue | CD83 |
| blue | CD84 |
| pink | CD86 |
| blue | CD8A |
| blue | CD8B |
| brown | CD93 |
| blue | CD96 |
| blue | CD97 |
| magenta | CD99L2 |
| brown | CDAN1 |
| blue | CDC16 |
| green | CDC34 |
| green | CDC37 |
| pink | CDC42BPG |
| brown | CDC42EP3 |
| yellow | CDC42EP4 |
| brown | CDC42EP5 |
| blue | CDC42SE2 |
| magenta | CDC42 |
| brown | CDCA5 |
| pink | CDCA7L |
| brown | CDH11 |
| brown | CDH13 |
| yellow | CDH23 |
| magenta | CDH26 |
| grey | CDH3 |
| brown | CDH5 |
| grey | CDHR1 |
| green | CDIPT |
| red | CDK10 |
| brown | CDK11A |
| blue | CDK16 |
| yellow | CDK18 |
| brown | CDK1 |
| red | CDK3 |
| green | CDK4 |
| grey | CDK5RAP2 |
| red | CDK5RAP3 |
| magenta | CDKN1A |
| blue | CDKN1B |
| blue | CDKN1C |
| brown | CDKN2A |
| magenta | CDKN2B |
| brown | CDKN2C |
| yellow | CDON |
| blue | CDR2L |
| blue | CDRT4 |
| magenta | CDS1 |
| grey | CDS2 |
| green | CDT1 |
| magenta | CEACAM1 |
| magenta | CEACAM5 |
| magenta | CEACAM6 |
| magenta | CEACAM7 |
| green | CEBPD |
| brown | CEBPG |
| pink | CECR1 |
| green | CECR5 |
| blue | CELF2 |
| blue | CEL |
| brown | CENPA |
| brown | CENPQ |
| red | CENPT |
| green | CENPV |
| yellow | CEP135 |
| grey | CEP250 |
| brown | CEP72 |
| brown | CERCAM |
| brown | CERK |
| brown | CES3 |
| green | CFD |
| brown | CFI |
| yellow | CFLAR |
| blue | CFP |
| yellow | CGNL1 |
| magenta | CGN |
| yellow | CGRRF1 |
| brown | CH25H |
| yellow | CHAC2 |
| brown | CHAF1A |
| green | CHCHD3 |
| grey | CHDH |
| pink | CHEK1 |
| pink | CHI3L1 |
| blue | CHI3L2 |
| pink | CHIT1 |
| red | CHKB.CPT1B |
| pink | CHMP4C |
| blue | CHMP7 |
| brown | CHN1 |
| grey | CHP2 |
| brown | CHPF2 |
| brown | CHPF |
| yellow | CHPT1 |
| brown | CHRDL1 |
| brown | CHRD |
| green | CHST10 |
| pink | CHST11 |
| yellow | CHST14 |
| yellow | CHST15 |
| brown | CHST1 |
| blue | CHST2 |
| blue | CHST6 |
| green | CHST7 |
| red | CHTF18 |
| brown | CIDEB |
| blue | CIITA |
| brown | CILP2 |
| blue | CISH |
| blue | CITED2 |
| yellow | CIZ1 |
| brown | CKAP4 |
| yellow | CKMT1B |
| magenta | CLCA2 |
| magenta | CLCA4 |
| blue | CLCF1 |
| brown | CLCN4 |
| blue | CLCN6 |
| yellow | CLDN10 |
| blue | CLDN15 |
| magenta | CLDN23 |
| yellow | CLDN3 |
| magenta | CLDN4 |
| brown | CLDN7 |
| blue | CLEC10A |
| brown | CLEC11A |
| brown | CLEC14A |
| yellow | CLEC1A |
| blue | CLEC2D |
| brown | CLEC3B |
| pink | CLEC5A |
| pink | CLEC7A |
| yellow | CLGN |
| blue | CLIC2 |
| blue | CLIC5 |
| yellow | CLIP2 |
| yellow | CLIP3 |
| green | CLIP4 |
| red | CLK1 |
| red | CLK2 |
| yellow | CLN5 |
| grey | CLN8 |
| grey | CLNS1A |
| brown | CLP1 |
| green | CLPP |
| blue | CLSTN3 |
| green | CLTA |
| brown | CLU |
| brown | CMAH |
| grey | CMAS |
| grey | CMBL |
| pink | CMKLR1 |
| pink | CMTM3 |
| yellow | CMTM4 |
| blue | CMTM7 |
| pink | CNDP2 |
| brown | CNN1 |
| green | CNN2 |
| green | CNN3 |
| grey | CNNM2 |
| green | CNOT3 |
| brown | CNOT8 |
| brown | CNRIP1 |
| brown | CNTD1 |
| yellow | CNTNAP2 |
| brown | CNTROB |
| grey | COCH |
| green | COG3 |
| grey | COG7 |
| brown | COL10A1 |
| brown | COL11A1 |
| brown | COL12A1 |
| brown | COL14A1 |
| brown | COL15A1 |
| yellow | COL16A1 |
| yellow | COL18A1 |
| yellow | COL19A1 |
| brown | COL1A1 |
| brown | COL1A2 |
| yellow | COL22A1 |
| yellow | COL23A1 |
| green | COL27A1 |
| brown | COL3A1 |
| brown | COL4A1 |
| brown | COL4A2 |
| yellow | COL4A4 |
| brown | COL5A1 |
| brown | COL5A2 |
| brown | COL5A3 |
| brown | COL6A1 |
| brown | COL6A2 |
| brown | COL6A3 |
| brown | COL8A1 |
| brown | COLEC12 |
| yellow | COMMD10 |
| brown | COMP |
| green | COPE |
| brown | COPS3 |
| green | COPS5 |
| blue | COPS7A |
| green | COQ5 |
| brown | COQ7 |
| blue | CORO1A |
| blue | CORO7 |
| blue | COTL1 |
| grey | COX10 |
| green | COX11 |
| grey | COX15 |
| green | COX4I1 |
| green | COX5A |
| yellow | COX6B2 |
| brown | CPA3 |
| yellow | CPAMD8 |
| blue | CPEB1 |
| green | CPEB2 |
| green | CPE |
| green | CPM |
| yellow | CPNE2 |
| blue | CPNE5 |
| pink | CPNE7 |
| red | CPT1B |
| grey | CPT2 |
| pink | CPVL |
| brown | CPXM1 |
| brown | CPXM2 |
| brown | CPZ |
| blue | CR1 |
| blue | CR2 |
| grey | CRAT |
| yellow | CRB2 |
| green | CRB3 |
| grey | CRBN |
| blue | CRCP |
| yellow | CREB3L1 |
| green | CREB5 |
| blue | CREBL2 |
| pink | CREG1 |
| yellow | CREM |
| blue | CRISPLD1 |
| brown | CRISPLD2 |
| grey | CRMP1 |
| red | CROCCL1 |
| green | CROCC |
| blue | CRTAM |
| green | CRTC1 |
| brown | CRY2 |
| green | CRYZ |
| red | CSAD |
| pink | CSF1R |
| blue | CSF1 |
| blue | CSF2RA |
| blue | CSF2RB |
| pink | CSF3R |
| brown | CSGALNACT1 |
| green | CSGALNACT2 |
| blue | CSK |
| green | CSNK1D |
| blue | CSNK1E |
| green | CSNK1G2 |
| brown | CSPG4 |
| brown | CST1 |
| blue | CST7 |
| magenta | CSTB |
| blue | CTBP2 |
| green | CTDP1 |
| brown | CTGF |
| brown | CTHRC1 |
| blue | CTLA4 |
| yellow | CTNNAL1 |
| blue | CTNS |
| yellow | CTPS |
| pink | CTSB |
| pink | CTSC |
| pink | CTSD |
| yellow | CTSE |
| pink | CTSH |
| brown | CTSK |
| pink | CTSL1 |
| green | CTSO |
| pink | CTSS |
| blue | CTSW |
| pink | CTSZ |
| blue | CTTN |
| green | CTU1 |
| green | CTXN1 |
| blue | CUEDC1 |
| brown | CUL7 |
| grey | CUL9 |
| brown | CUX1 |
| brown | CWC25 |
| brown | CWC27 |
| blue | CX3CL1 |
| blue | CX3CR1 |
| brown | CXCL12 |
| blue | CXCL13 |
| magenta | CXCL17 |
| blue | CXCL1 |
| yellow | CXCL2 |
| grey | CXCL6 |
| blue | CXCL9 |
| blue | CXCR2P1 |
| blue | CXCR3 |
| blue | CXCR4 |
| blue | CXCR5 |
| blue | CXCR6 |
| red | CXXC1 |
| brown | CXXC5 |
| brown | CXorf36 |
| green | CXorf57 |
| grey | CYB561D1 |
| grey | CYB5A |
| blue | CYB5R2 |
| brown | CYB5R3 |
| blue | CYBASC3 |
| pink | CYBB |
| blue | CYFIP2 |
| yellow | CYGB |
| brown | CYP26B1 |
| yellow | CYP27A1 |
| grey | CYP27C1 |
| magenta | CYP2C18 |
| brown | CYP2R1 |
| blue | CYP2S1 |
| brown | CYP2U1 |
| grey | CYP4F11 |
| grey | CYP4F3 |
| blue | CYP4V2 |
| yellow | CYP4X1 |
| blue | CYP51A1 |
| brown | CYR61 |
| blue | CYTH4 |
| blue | CYTIP |
| blue | CYTSB |
| brown | CYYR1 |
| grey | CYorf15B |
| red | D2HGDH |
| grey | D4S234E |
| brown | DAAM2 |
| grey | DAB2IP |
| brown | DAB2 |
| brown | DACT1 |
| yellow | DACT2 |
| brown | DAPK1 |
| green | DAPK3 |
| magenta | DAPP1 |
| brown | DAP |
| blue | DARC |
| green | DAZAP1 |
| brown | DBF4 |
| blue | DBN1 |
| grey | DCAF11 |
| green | DCAF15 |
| brown | DCAF8 |
| grey | DCAKD |
| brown | DCHS1 |
| green | DCI |
| brown | DCLK1 |
| brown | DCLRE1C |
| brown | DCN |
| blue | DCP2 |
| green | DCTD |
| red | DCTN1 |
| green | DCTN2 |
| brown | DCTN6 |
| green | DCTPP1 |
| pink | DCUN1D4 |
| grey | DDIT4 |
| brown | DDR2 |
| grey | DDX10 |
| red | DDX11 |
| red | DDX12 |
| blue | DDX1 |
| grey | DDX23 |
| green | DDX39 |
| grey | DDX3Y |
| grey | DDX47 |
| green | DDX49 |
| green | DDX54 |
| red | DDX55 |
| green | DDX59 |
| blue | DEDD |
| blue | DEF6 |
| blue | DEGS1 |
| grey | DEGS2 |
| brown | DEM1 |
| blue | DENND1C |
| blue | DENND2D |
| blue | DENND3 |
| blue | DENND4B |
| brown | DENND5B |
| yellow | DENR |
| yellow | DEPDC7 |
| blue | DERA |
| blue | DERL3 |
| grey | DET1 |
| grey | DFFA |
| blue | DFNA5 |
| yellow | DFNB31 |
| yellow | DGAT2 |
| blue | DGCR2 |
| magenta | DGKA |
| brown | DGKD |
| magenta | DHCR24 |
| blue | DHCR7 |
| grey | DHDDS |
| green | DHPS |
| green | DHRS11 |
| magenta | DHRS9 |
| blue | DHX32 |
| red | DHX34 |
| green | DHX37 |
| brown | DIO2 |
| grey | DIS3L2 |
| brown | DIXDC1 |
| blue | DKFZP586I1420 |
| grey | DKK1 |
| brown | DKK3 |
| brown | DLC1 |
| grey | DLD |
| brown | DLEU2 |
| brown | DLG3 |
| brown | DLG4 |
| brown | DLGAP4 |
| yellow | DLK2 |
| yellow | DLL1 |
| brown | DLL4 |
| grey | DLX5 |
| blue | DLX6 |
| yellow | DMD |
| green | DMRTA1 |
| brown | DMRTA2 |
| pink | DMXL2 |
| yellow | DNAH11 |
| grey | DNAH17 |
| blue | DNAH1 |
| grey | DNAH5 |
| grey | DNAJA3 |
| brown | DNAJB5 |
| grey | DNAJB6 |
| brown | DNAJB9 |
| yellow | DNAJC18 |
| grey | DNAJC21 |
| green | DNAJC24 |
| red | DNAJC25 |
| blue | DNASE1L3 |
| green | DNASE2 |
| green | DNM2 |
| blue | DOCK10 |
| blue | DOCK11 |
| green | DOCK1 |
| blue | DOCK2 |
| blue | DOCK6 |
| blue | DOCK8 |
| green | DOHH |
| pink | DOK1 |
| blue | DOK2 |
| blue | DOK3 |
| blue | DOK4 |
| brown | DONSON |
| green | DOT1L |
| green | DPH1 |
| green | DPH2 |
| green | DPP3 |
| brown | DPP4 |
| green | DPP9 |
| brown | DPT |
| green | DPY19L1 |
| yellow | DPYSL2 |
| brown | DPYSL3 |
| magenta | DQX1 |
| yellow | DRAM1 |
| green | DSC2 |
| brown | DSCR6 |
| brown | DSEL |
| red | DSE |
| magenta | DSG3 |
| blue | DSTN |
| blue | DTX1 |
| brown | DTX2 |
| red | DTX3 |
| grey | DTX4 |
| magenta | DUOX1 |
| magenta | DUOX2 |
| magenta | DUOXA1 |
| magenta | DUOXA2 |
| green | DUS3L |
| grey | DUS4L |
| green | DUSP10 |
| blue | DUSP14 |
| magenta | DUSP22 |
| blue | DUSP2 |
| blue | DUSP4 |
| grey | DUSP5 |
| magenta | DUSP7 |
| blue | DUSP9 |
| brown | DVL2 |
| blue | DYNLT3 |
| yellow | DYRK1B |
| brown | DYSF |
| blue | DZIP1L |
| yellow | DZIP1 |
| brown | E2F2 |
| green | EBAG9 |
| brown | EBF1 |
| yellow | EBF3 |
| blue | EBI3 |
| yellow | ECE1 |
| red | ECHDC2 |
| blue | ECHDC3 |
| brown | ECM2 |
| green | ECSIT |
| yellow | EDARADD |
| red | EDIL3 |
| grey | EDN1 |
| grey | EDN2 |
| brown | EDNRA |
| brown | EDNRB |
| brown | EEPD1 |
| brown | EFEMP1 |
| brown | EFEMP2 |
| red | EFHC1 |
| yellow | EFHD1 |
| grey | EFHD2 |
| blue | EFNA1 |
| magenta | EFNA3 |
| blue | EFNA5 |
| grey | EFNB1 |
| green | EFNB2 |
| brown | EFS |
| grey | EFTUD2 |
| red | EGFL8 |
| yellow | EGFLAM |
| red | EHD2 |
| brown | EHD3 |
| brown | EID1 |
| grey | EIF1AY |
| grey | EIF2AK1 |
| green | EIF3CL |
| green | EIF3G |
| blue | EIF4E3 |
| green | EIF4EBP1 |
| grey | EIF4E |
| green | ELAVL1 |
| magenta | ELF3 |
| green | ELL |
| blue | ELMO1 |
| red | ELMOD3 |
| brown | ELN |
| green | ELOF1 |
| blue | ELOVL4 |
| blue | ELOVL6 |
| yellow | ELP2 |
| green | ELP3 |
| brown | ELP4 |
| brown | ELTD1 |
| blue | EMB |
| brown | EMCN |
| red | EME1 |
| brown | EMILIN1 |
| pink | EMILIN2 |
| brown | EML1 |
| magenta | EMP1 |
| magenta | EMP2 |
| pink | EMP3 |
| pink | EMR2 |
| yellow | EN2 |
| brown | ENC1 |
| red | ENDOD1 |
| green | ENDOG |
| red | ENGASE |
| brown | ENG |
| brown | ENOPH1 |
| red | ENOSF1 |
| brown | ENPEP |
| brown | ENPP1 |
| blue | ENPP2 |
| green | ENPP5 |
| yellow | ENPP6 |
| blue | ENTPD1 |
| blue | EOMES |
| magenta | EPB41L1 |
| pink | EPB41L3 |
| grey | EPB41L4B |
| blue | EPB49 |
| blue | EPCAM |
| brown | EPDR1 |
| magenta | EPHA1 |
| magenta | EPHA2 |
| brown | EPHA3 |
| yellow | EPHB1 |
| yellow | EPHB2 |
| magenta | EPHB3 |
| grey | EPHB4 |
| grey | EPHB6 |
| brown | EPHX3 |
| magenta | EPN2 |
| magenta | EPN3 |
| magenta | EPS8L1 |
| magenta | EPS8L2 |
| blue | ERAP2 |
| pink | ERBB2 |
| brown | ERCC3 |
| blue | ERCC5 |
| brown | ERF |
| red | ERGIC1 |
| green | ERICH1 |
| brown | ERLEC1 |
| yellow | ERO1LB |
| green | ERRFI1 |
| brown | ESAM |
| yellow | ESR1 |
| blue | ESRP1 |
| red | ESYT1 |
| grey | ETF1 |
| green | ETFA |
| blue | ETS1 |
| yellow | ETV1 |
| brown | ETV5 |
| yellow | ETV6 |
| blue | EVI2A |
| blue | EVI2B |
| yellow | EVI5L |
| blue | EVL |
| magenta | EVPL |
| green | EXOC1 |
| blue | EXOC6 |
| grey | EXOC7 |
| brown | EXOSC10 |
| green | EXOSC3 |
| red | EXT1 |
| brown | EXT2 |
| red | EXTL3 |
| brown | EYA2 |
| brown | EZH2 |
| blue | F11R |
| brown | F13A1 |
| brown | F2RL2 |
| brown | F2R |
| blue | F3 |
| blue | F5 |
| grey | FA2H |
| yellow | FAAH2 |
| magenta | FABP5 |
| green | FADD |
| magenta | FADS1 |
| grey | FADS2 |
| blue | FAIM3 |
| brown | FAIM |
| brown | FAM101B |
| grey | FAM104A |
| blue | FAM105A |
| yellow | FAM105B |
| blue | FAM107A |
| blue | FAM107B |
| green | FAM108A1 |
| brown | FAM108C1 |
| brown | FAM110B |
| brown | FAM111A |
| blue | FAM113B |
| yellow | FAM117A |
| yellow | FAM117B |
| green | FAM125A |
| blue | FAM125B |
| blue | FAM129B |
| green | FAM149B1 |
| red | FAM156A |
| grey | FAM160A2 |
| blue | FAM160B1 |
| yellow | FAM161A |
| yellow | FAM164A |
| yellow | FAM167A |
| yellow | FAM171A1 |
| brown | FAM171B |
| grey | FAM174A |
| brown | FAM174B |
| brown | FAM176A |
| green | FAM188A |
| green | FAM188B |
| yellow | FAM189A2 |
| blue | FAM189B |
| yellow | FAM18B2 |
| red | FAM193B |
| green | FAM195A |
| brown | FAM198B |
| green | FAM200A |
| brown | FAM20A |
| blue | FAM21B |
| pink | FAM26F |
| green | FAM32A |
| grey | FAM35A |
| grey | FAM35B |
| blue | FAM3C |
| yellow | FAM45A |
| green | FAM46A |
| blue | FAM46C |
| green | FAM48A |
| blue | FAM49A |
| grey | FAM49B |
| green | FAM50B |
| blue | FAM53B |
| brown | FAM54A |
| brown | FAM55C |
| brown | FAM57A |
| grey | FAM65A |
| blue | FAM65B |
| yellow | FAM65C |
| yellow | FAM70A |
| brown | FAM72B |
| red | FAM73B |
| grey | FAM76A |
| blue | FAM78A |
| brown | FAM81A |
| magenta | FAM83A |
| magenta | FAM83C |
| brown | FAM83D |
| brown | FAM83E |
| blue | FAM83H |
| grey | FAM86C |
| blue | FAM89A |
| magenta | FAM92A1 |
| red | FAM98A |
| brown | FANCA |
| brown | FANCD2 |
| brown | FANCE |
| pink | FANCF |
| green | FANCG |
| red | FANCL |
| brown | FAP |
| green | FARSA |
| yellow | FASN |
| brown | FASTKD1 |
| red | FASTKD3 |
| yellow | FAS |
| brown | FBF1 |
| brown | FBLN2 |
| brown | FBLN5 |
| grey | FBLN7 |
| brown | FBN1 |
| pink | FBP1 |
| green | FBRSL1 |
| grey | FBRS |
| green | FBXL12 |
| grey | FBXL18 |
| grey | FBXL19 |
| brown | FBXL7 |
| grey | FBXO18 |
| green | FBXO25 |
| yellow | FBXO2 |
| grey | FBXO41 |
| magenta | FBXO42 |
| green | FBXO46 |
| green | FBXO8 |
| green | FBXW7 |
| green | FBXW9 |
| blue | FCER1A |
| pink | FCER1G |
| yellow | FCGBP |
| pink | FCGR1A |
| pink | FCGR1B |
| pink | FCGR2A |
| pink | FCGR2B |
| pink | FCGR3A |
| blue | FCHO1 |
| red | FCHSD1 |
| blue | FCRL5 |
| blue | FCRLA |
| yellow | FDFT1 |
| grey | FECH |
| red | FER1L4 |
| brown | FERMT2 |
| blue | FERMT3 |
| pink | FES |
| grey | FEZ1 |
| grey | FEZ2 |
| blue | FGD2 |
| blue | FGD3 |
| brown | FGD5 |
| blue | FGF11 |
| yellow | FGF1 |
| yellow | FGF2 |
| blue | FGFBP1 |
| brown | FGFR1 |
| brown | FGFR3 |
| brown | FGFRL1 |
| grey | FGGY |
| pink | FGL2 |
| pink | FGR |
| green | FHDC1 |
| yellow | FHOD3 |
| grey | FIG4 |
| brown | FILIP1L |
| brown | FIP1L1 |
| green | FIZ1 |
| yellow | FJX1 |
| brown | FKBP10 |
| blue | FKBP11 |
| grey | FKBP4 |
| blue | FKBP5 |
| brown | FKBP7 |
| green | FKBP8 |
| brown | FKBP9 |
| grey | FKRP |
| blue | FLI1 |
| grey | FLII |
| brown | FLJ10357 |
| grey | FLJ33630 |
| blue | FLJ40330 |
| red | FLJ45445 |
| blue | FLJ90757 |
| brown | FLRT2 |
| red | FLRT3 |
| brown | FLT1 |
| brown | FLT4 |
| pink | FLVCR2 |
| blue | FMNL1 |
| blue | FMNL3 |
| brown | FMO1 |
| brown | FMO2 |
| brown | FMOD |
| brown | FN1 |
| grey | FN3KRP |
| green | FN3K |
| blue | FNBP1 |
| brown | FNDC1 |
| grey | FOLH1 |
| pink | FOLR2 |
| blue | FOSL1 |
| grey | FOXA1 |
| green | FOXC1 |
| blue | FOXD1 |
| brown | FOXF1 |
| brown | FOXF2 |
| grey | FOXJ1 |
| green | FOXK2 |
| yellow | FOXN1 |
| brown | FOXP1 |
| blue | FOXP3 |
| yellow | FOXP4 |
| grey | FOXQ1 |
| pink | FPR1 |
| pink | FPR3 |
| yellow | FRMD4A |
| blue | FRMD8 |
| brown | FRY |
| blue | FRZB |
| blue | FSCN1 |
| brown | FSTL1 |
| brown | FSTL3 |
| yellow | FSTL4 |
| brown | FST |
| pink | FTL |
| blue | FTSJ1 |
| green | FTSJ3 |
| green | FUBP1 |
| blue | FUCA1 |
| yellow | FUCA2 |
| magenta | FUT2 |
| magenta | FUT3 |
| yellow | FUT4 |
| magenta | FUT6 |
| grey | FXC1 |
| green | FXN |
| blue | FXYD5 |
| yellow | FXYD6 |
| blue | FYB |
| blue | FYN |
| brown | FZD10 |
| brown | FZD4 |
| yellow | FZD7 |
| yellow | FZD8 |
| green | FZR1 |
| brown | G0S2 |
| blue | GAB3 |
| brown | GABARAPL1 |
| red | GABBR1 |
| grey | GABRP |
| magenta | GABRQ |
| green | GADD45GIP1 |
| green | GALC |
| magenta | GALE |
| blue | GALM |
| yellow | GALNT11 |
| red | GALNT2 |
| brown | GALNT6 |
| yellow | GALNTL4 |
| brown | GAS1 |
| brown | GAS7 |
| brown | GATA2 |
| blue | GATA3 |
| yellow | GATA6 |
| grey | GATM |
| red | GBA2 |
| grey | GBAS |
| grey | GBA |
| pink | GBGT1 |
| blue | GBP3 |
| blue | GBP4 |
| blue | GCA |
| green | GCDH |
| yellow | GCET2 |
| brown | GCH1 |
| blue | GCNT1 |
| grey | GCNT3 |
| pink | GDA |
| grey | GDE1 |
| green | GDF11 |
| blue | GDF15 |
| brown | GEFT |
| brown | GEN1 |
| blue | GFI1 |
| magenta | GFOD2 |
| yellow | GFPT2 |
| brown | GFRA1 |
| yellow | GGA2 |
| red | GGCX |
| grey | GGPS1 |
| yellow | GGT1 |
| brown | GGT5 |
| magenta | GGT6 |
| pink | GGTA1 |
| green | GHITM |
| red | GIGYF1 |
| blue | GIMAP1 |
| blue | GIMAP2 |
| blue | GIMAP4 |
| blue | GIMAP5 |
| blue | GIMAP6 |
| blue | GIMAP7 |
| blue | GIMAP8 |
| brown | GIN1 |
| green | GINS2 |
| yellow | GINS3 |
| magenta | GIPC1 |
| yellow | GIPC2 |
| green | GIT1 |
| blue | GIT2 |
| red | GJA1 |
| yellow | GJA3 |
| brown | GJA4 |
| brown | GJA5 |
| brown | GJB3 |
| yellow | GJB4 |
| magenta | GJB5 |
| brown | GJC1 |
| blue | GJD3 |
| brown | GK |
| grey | GLB1L |
| blue | GLCCI1 |
| brown | GLE1 |
| brown | GLI2 |
| green | GLI3 |
| pink | GLIPR1 |
| pink | GLIPR2 |
| brown | GLIS2 |
| brown | GLIS3 |
| brown | GLOD4 |
| blue | GLRX |
| red | GLS2 |
| green | GLS |
| grey | GLT25D1 |
| brown | GLT8D2 |
| green | GLTSCR1 |
| grey | GLUD1 |
| pink | GM2A |
| brown | GMEB2 |
| blue | GMFG |
| blue | GMIP |
| pink | GMPR |
| green | GNA12 |
| magenta | GNA15 |
| pink | GNAI2 |
| brown | GNAO1 |
| red | GNB1 |
| brown | GNB5 |
| brown | GNG11 |
| blue | GNG2 |
| blue | GNG7 |
| green | GNPDA1 |
| blue | GNPNAT1 |
| red | GNRHR2 |
| red | GNS |
| red | GOLGA2B |
| magenta | GOLGA6L10 |
| magenta | GOLGA6L9 |
| brown | GOLGA7B |
| red | GOLGA8A |
| red | GOLGA8B |
| blue | GOLM1 |
| yellow | GORAB |
| brown | GOSR2 |
| green | GOT1 |
| blue | GPAT2 |
| brown | GPATCH1 |
| blue | GPC1 |
| red | GPC2 |
| brown | GPC4 |
| brown | GPC6 |
| magenta | GPCPD1 |
| grey | GPER |
| green | GPI |
| green | GPN3 |
| yellow | GPNMB |
| green | GPR108 |
| brown | GPR109A |
| brown | GPR109B |
| magenta | GPR110 |
| blue | GPR114 |
| magenta | GPR115 |
| brown | GPR116 |
| brown | GPR124 |
| blue | GPR132 |
| brown | GPR137B |
| brown | GPR137C |
| blue | GPR153 |
| blue | GPR155 |
| yellow | GPR160 |
| brown | GPR161 |
| blue | GPR171 |
| red | GPR172B |
| red | GPR176 |
| blue | GPR183 |
| blue | GPR34 |
| yellow | GPR39 |
| brown | GPR4 |
| blue | GPR56 |
| blue | GPR65 |
| grey | GPR68 |
| blue | GPR87 |
| green | GPR98 |
| blue | GPRC5A |
| brown | GPRC5B |
| grey | GPRC5C |
| yellow | GPRIN2 |
| blue | GPSM3 |
| brown | GPX3 |
| grey | GPX7 |
| brown | GPX8 |
| yellow | GRAMD1A |
| yellow | GRAMD3 |
| yellow | GRAMD4 |
| blue | GRAP2 |
| blue | GRAP |
| blue | GRB2 |
| brown | GRB7 |
| brown | GREM1 |
| magenta | GRHL1 |
| magenta | GRHL3 |
| yellow | GRIN2A |
| green | GRIN2C |
| blue | GRIN2D |
| brown | GRK5 |
| brown | GRPEL2 |
| red | GRSF1 |
| brown | GRTP1 |
| green | GRWD1 |
| red | GSDMB |
| magenta | GSDMC |
| green | GSK3A |
| brown | GSPT2 |
| green | GSS |
| blue | GSTM1 |
| grey | GSTT1 |
| brown | GTDC1 |
| green | GTF2F1 |
| grey | GTF2H2B |
| blue | GTF2IRD1 |
| pink | GTF2IRD2B |
| red | GTF2IRD2P1 |
| red | GTPBP3 |
| blue | GTPBP4 |
| brown | GUCY1A3 |
| yellow | GUCY1B3 |
| red | GUSBP1 |
| blue | GVIN1 |
| brown | GYG2 |
| yellow | GYLTL1B |
| blue | GYPC |
| magenta | GZF1 |
| blue | GZMA |
| blue | GZMB |
| blue | GZMH |
| blue | GZMK |
| blue | GZMM |
| blue | H19 |
| grey | H2AFV |
| yellow | H2AFY2 |
| blue | HAAO |
| blue | HADHB |
| brown | HADH |
| pink | HAPLN3 |
| brown | HAT1 |
| brown | HAUS3 |
| red | HAUS5 |
| green | HAUS8 |
| pink | HAVCR2 |
| grey | HBA2 |
| grey | HBB |
| pink | HCG11 |
| blue | HCK |
| blue | HCLS1 |
| red | HCN3 |
| blue | HCST |
| magenta | HDAC1 |
| grey | HDAC2 |
| green | HDGFRP2 |
| grey | HDHD1A |
| blue | HDHD2 |
| brown | HDLBP |
| grey | HECTD3 |
| red | HEG1 |
| green | HELQ |
| brown | HEPH |
| green | HERC4 |
| blue | HERPUD1 |
| blue | HES1 |
| magenta | HES2 |
| pink | HEXA |
| red | HEXDC |
| yellow | HEY1 |
| grey | HEY2 |
| brown | HEYL |
| green | HGS |
| blue | HHEX |
| green | HIBCH |
| brown | HIC1 |
| green | HIST1H1C |
| grey | HIST1H2AC |
| grey | HIST1H2BJ |
| yellow | HIVEP3 |
| red | HK1 |
| pink | HK3 |
| blue | HKR1 |
| blue | HLA.DMA |
| pink | HLA.DMB |
| blue | HLA.DOA |
| blue | HLA.DOB |
| pink | HLA.DPA1 |
| pink | HLA.DPB1 |
| pink | HLA.DQA1 |
| blue | HLA.DQA2 |
| pink | HLA.DQB1 |
| blue | HLA.DQB2 |
| pink | HLA.DRA |
| pink | HLA.DRB1 |
| pink | HLA.DRB5 |
| blue | HLA.DRB6 |
| yellow | HLF |
| blue | HLX |
| brown | HMCN1 |
| green | HMG20B |
| blue | HMGA2 |
| brown | HMGB2 |
| red | HMGN1 |
| pink | HMGN5 |
| blue | HMHA1 |
| pink | HMOX1 |
| pink | HN1L |
| pink | HNMT |
| brown | HNRNPA2B1 |
| brown | HNRNPF |
| red | HNRNPH1 |
| red | HNRNPL |
| green | HNRNPM |
| red | HNRPDL |
| magenta | HOMER2 |
| brown | HOMEZ |
| red | HOOK2 |
| grey | HOXA10 |
| grey | HOXA3 |
| yellow | HOXB13 |
| brown | HOXB2 |
| yellow | HOXC6 |
| grey | HOXD10 |
| grey | HOXD11 |
| grey | HOXD13 |
| brown | HPGD |
| green | HPS4 |
| brown | HR |
| magenta | HS3ST1 |
| pink | HS3ST3A1 |
| blue | HS3ST4 |
| pink | HS6ST1 |
| yellow | HS6ST2 |
| brown | HSD17B11 |
| blue | HSD17B12 |
| pink | HSD17B14 |
| red | HSF4 |
| blue | HSH2D |
| brown | HSPA12B |
| brown | HSPA14 |
| grey | HSPA1A |
| green | HSPA1B |
| green | HSPA2 |
| red | HSPA4 |
| green | HSPA6 |
| red | HSPA8 |
| brown | HSPA9 |
| yellow | HSPB8 |
| green | HSPBP1 |
| green | HSPD1 |
| grey | HSPH1 |
| brown | HTRA1 |
| brown | HTRA3 |
| yellow | HUNK |
| green | HUS1 |
| blue | HVCN1 |
| blue | HYAL1 |
| brown | HYOU1 |
| yellow | ICAM1 |
| blue | ICAM2 |
| blue | ICAM3 |
| yellow | ICAM5 |
| grey | ICMT |
| yellow | ICOSLG |
| blue | ICOS |
| blue | ID2 |
| grey | IDH3A |
| magenta | IDI1 |
| green | IDS |
| green | IER2 |
| blue | IER3 |
| pink | IFFO1 |
| pink | IFI30 |
| pink | IFITM2 |
| blue | IFNAR2 |
| green | IFNGR1 |
| brown | IFRD1 |
| grey | IFT74 |
| yellow | IFT88 |
| brown | IGF2 |
| blue | IGFBP2 |
| brown | IGFBP3 |
| brown | IGFBP4 |
| brown | IGFBP5 |
| brown | IGFBP7 |
| blue | IGHMBP2 |
| blue | IGJ |
| pink | IGSF6 |
| grey | IKBIP |
| blue | IKBKB |
| yellow | IKBKE |
| blue | IKZF1 |
| blue | IL10RA |
| red | IL11RA |
| blue | IL12RB1 |
| red | IL13RA1 |
| yellow | IL15 |
| blue | IL16 |
| red | IL17RB |
| yellow | IL17REL |
| magenta | IL17RE |
| blue | IL18BP |
| yellow | IL18R1 |
| pink | IL18 |
| green | IL1A |
| grey | IL1B |
| brown | IL1R1 |
| yellow | IL1R2 |
| brown | IL20RA |
| magenta | IL20RB |
| blue | IL21R |
| yellow | IL23A |
| yellow | IL27RA |
| blue | IL2RA |
| blue | IL2RB |
| blue | IL2RG |
| pink | IL32 |
| blue | IL3RA |
| pink | IL4I1 |
| grey | IL4R |
| yellow | IL7 |
| blue | IL8 |
| brown | ILDR1 |
| red | ILF3 |
| green | ILVBL |
| green | IMMT |
| grey | INA |
| yellow | ING1 |
| red | ING5 |
| brown | INHBA |
| brown | INHBB |
| brown | INMT |
| yellow | INPP1 |
| yellow | INPP4A |
| blue | INPP5A |
| blue | INPP5B |
| blue | INPP5D |
| red | INPP5E |
| grey | INPP5F |
| green | INSIG2 |
| yellow | INTS12 |
| green | INTS5 |
| green | INTS9 |
| green | INTU |
| blue | IPCEF1 |
| brown | IPO13 |
| grey | IPO4 |
| magenta | IPPK |
| red | IQCC |
| yellow | IQCE |
| brown | IQCG |
| pink | IQGAP2 |
| yellow | IRAK2 |
| blue | IRAK4 |
| blue | IRF1 |
| green | IRF2 |
| blue | IRF4 |
| pink | IRF5 |
| blue | IRF8 |
| yellow | IRS2 |
| brown | IRX4 |
| yellow | IRX5 |
| yellow | ISL1 |
| brown | ISLR |
| brown | ISOC1 |
| brown | ITGA11 |
| brown | ITGA1 |
| blue | ITGA4 |
| brown | ITGA5 |
| blue | ITGAE |
| blue | ITGAL |
| yellow | ITGAM |
| green | ITGAV |
| pink | ITGAX |
| red | ITGB1 |
| pink | ITGB2 |
| red | ITGB3BP |
| brown | ITGB3 |
| grey | ITGB4 |
| blue | ITGB5 |
| green | ITGB6 |
| blue | ITGB7 |
| brown | ITGBL1 |
| brown | ITIH5 |
| blue | ITK |
| blue | ITM2A |
| grey | ITM2B |
| blue | ITM2C |
| magenta | ITPKC |
| blue | ITPR1 |
| green | ITPRIPL1 |
| brown | IVNS1ABP |
| blue | IWS1 |
| yellow | JAG2 |
| blue | JAK2 |
| blue | JAK3 |
| brown | JAM3 |
| yellow | JAZF1 |
| blue | JMJD5 |
| red | JMJD7.PLA2G4B |
| blue | JSRP1 |
| grey | JUB |
| green | JUNB |
| green | JUND |
| magenta | JUP |
| brown | KAL1 |
| brown | KALRN |
| brown | KATNA1 |
| magenta | KAZ |
| green | KBTBD2 |
| blue | KBTBD8 |
| blue | KCNAB2 |
| red | KCNC3 |
| yellow | KCNC4 |
| yellow | KCND1 |
| brown | KCNE4 |
| yellow | KCNIP3 |
| grey | KCNJ11 |
| red | KCNJ15 |
| yellow | KCNJ5 |
| brown | KCNJ8 |
| blue | KCNK1 |
| blue | KCNK5 |
| magenta | KCNK6 |
| yellow | KCNMA1 |
| brown | KCNN3 |
| blue | KCNN4 |
| grey | KCNQ1 |
| blue | KCNS1 |
| grey | KCNS3 |
| red | KCTD10 |
| blue | KCTD11 |
| pink | KCTD12 |
| red | KCTD13 |
| yellow | KCTD15 |
| blue | KDELC1 |
| blue | KDELR2 |
| brown | KDELR3 |
| blue | KDM1A |
| grey | KDM5D |
| brown | KDR |
| grey | KDSR |
| green | KEAP1 |
| yellow | KEL |
| grey | KHDRBS1 |
| grey | KHDRBS3 |
| green | KIAA0020 |
| blue | KIAA0040 |
| green | KIAA0114 |
| blue | KIAA0125 |
| grey | KIAA0141 |
| brown | KIAA0195 |
| green | KIAA0319L |
| grey | KIAA0391 |
| brown | KIAA0427 |
| yellow | KIAA0649 |
| green | KIAA0664 |
| blue | KIAA0748 |
| red | KIAA0895L |
| blue | KIAA0895 |
| red | KIAA0907 |
| blue | KIAA0922 |
| blue | KIAA1274 |
| blue | KIAA1279 |
| grey | KIAA1324 |
| brown | KIAA1462 |
| red | KIAA1529 |
| yellow | KIAA1543 |
| magenta | KIAA1609 |
| brown | KIAA1644 |
| red | KIAA1683 |
| green | KIAA1712 |
| pink | KIAA1841 |
| blue | KIAA1949 |
| green | KIAA1967 |
| grey | KIAA2022 |
| yellow | KIF21A |
| blue | KIF21B |
| brown | KIF26A |
| brown | KIF26B |
| brown | KIF2C |
| brown | KIF3C |
| yellow | KIFAP3 |
| brown | KIFC1 |
| red | KIFC2 |
| brown | KIN |
| blue | KLC2 |
| blue | KLC3 |
| green | KLF16 |
| blue | KLF2 |
| blue | KLF4 |
| yellow | KLHDC7B |
| red | KLHL17 |
| yellow | KLHL29 |
| green | KLHL2 |
| blue | KLHL6 |
| blue | KLRB1 |
| grey | KLRG2 |
| blue | KLRK1 |
| green | KRCC1 |
| yellow | KREMEN2 |
| green | KRI1 |
| blue | KRT10 |
| magenta | KRT13 |
| brown | KRT15 |
| blue | KRT17 |
| green | KRT18 |
| brown | KRT19 |
| brown | KRT24 |
| grey | KRT31 |
| brown | KRT5 |
| grey | KRT7 |
| green | KRT8 |
| yellow | KSR1 |
| grey | KYNU |
| yellow | L3MBTL4 |
| pink | LACTB |
| magenta | LAD1 |
| blue | LAG3 |
| pink | LAIR1 |
| brown | LAMA1 |
| brown | LAMA2 |
| brown | LAMA4 |
| brown | LAMB1 |
| brown | LAMB2 |
| grey | LAMB3 |
| grey | LAMP1 |
| blue | LAPTM4B |
| pink | LAPTM5 |
| yellow | LARGE |
| yellow | LARP6 |
| brown | LARP7 |
| magenta | LASS3 |
| blue | LAT2 |
| blue | LAT |
| blue | LAX1 |
| brown | LAYN |
| blue | LBH |
| blue | LCK |
| blue | LCLAT1 |
| magenta | LCN2 |
| blue | LCP1 |
| blue | LCP2 |
| brown | LDB2 |
| grey | LDHA |
| blue | LEF1 |
| yellow | LEMD1 |
| red | LENG8 |
| green | LENG9 |
| yellow | LEO1 |
| brown | LEPRE1 |
| brown | LEPREL2 |
| blue | LEPROTL1 |
| green | LEPR |
| blue | LETM1 |
| red | LETMD1 |
| blue | LFNG |
| blue | LGALS2 |
| blue | LGALS9 |
| yellow | LGI2 |
| magenta | LGI3 |
| pink | LGMN |
| brown | LGR5 |
| pink | LHFPL2 |
| grey | LHFPL4 |
| brown | LHFP |
| blue | LHX6 |
| yellow | LIFR |
| brown | LIF |
| red | LIG1 |
| pink | LILRB1 |
| pink | LILRB2 |
| pink | LILRB3 |
| pink | LILRB4 |
| brown | LIMCH1 |
| blue | LIMD2 |
| blue | LIME1 |
| red | LIMK1 |
| blue | LIMK2 |
| brown | LIMS2 |
| brown | LINS1 |
| pink | LIPA |
| green | LIPE |
| green | LIPG |
| magenta | LIPH |
| red | LIPT1 |
| yellow | LITAF |
| blue | LIX1L |
| blue | LLGL2 |
| brown | LMCD1 |
| blue | LMNA |
| green | LMNB2 |
| blue | LMO2 |
| brown | LMO4 |
| brown | LMOD1 |
| blue | LMTK3 |
| blue | LNP1 |
| pink | LNX1 |
| blue | LOC100125556 |
| brown | LOC100128191 |
| pink | LOC100129034 |
| red | LOC100129637 |
| green | LOC100130776 |
| red | LOC100132287 |
| red | LOC100133161 |
| red | LOC100133331 |
| grey | LOC100134229 |
| magenta | LOC100190939 |
| red | LOC100216545 |
| green | LOC113230 |
| red | LOC115110 |
| red | LOC146880 |
| red | LOC150776 |
| brown | LOC151162 |
| red | LOC162632 |
| red | LOC220594 |
| brown | LOC254559 |
| yellow | LOC283070 |
| yellow | LOC283174 |
| yellow | LOC283267 |
| red | LOC285074 |
| red | LOC338799 |
| red | LOC339047 |
| red | LOC349114 |
| blue | LOC374443 |
| brown | LOC387647 |
| magenta | LOC388152 |
| blue | LOC388692 |
| blue | LOC399744 |
| yellow | LOC399959 |
| red | LOC400027 |
| blue | LOC400657 |
| yellow | LOC401093 |
| blue | LOC401397 |
| green | LOC407835 |
| grey | LOC440173 |
| red | LOC440944 |
| green | LOC550112 |
| grey | LOC595101 |
| blue | LOC606724 |
| red | LOC642846 |
| grey | LOC654433 |
| yellow | LOC728392 |
| brown | LOC728554 |
| grey | LOC728613 |
| green | LOC729991.MEF2B |
| grey | LOC730101 |
| magenta | LOC80154 |
| brown | LOC81691 |
| yellow | LOC84740 |
| yellow | LOC84856 |
| grey | LOC90784 |
| red | LOC91316 |
| blue | LOC96610 |
| green | LONP1 |
| brown | LOXL1 |
| brown | LOXL2 |
| brown | LOXL3 |
| brown | LOXL4 |
| magenta | LPAR5 |
| pink | LPCAT1 |
| yellow | LPCAT4 |
| green | LPHN1 |
| green | LPHN2 |
| blue | LPIN1 |
| yellow | LPIN2 |
| red | LPIN3 |
| brown | LPL |
| blue | LPPR2 |
| blue | LPXN |
| grey | LRAT |
| red | LRDD |
| green | LRFN3 |
| magenta | LRG1 |
| brown | LRIG1 |
| blue | LRMP |
| red | LRP10 |
| blue | LRP11 |
| red | LRP1 |
| green | LRP3 |
| brown | LRRC15 |
| blue | LRRC1 |
| pink | LRRC25 |
| red | LRRC28 |
| brown | LRRC32 |
| blue | LRRC33 |
| brown | LRRC37B2 |
| blue | LRRC42 |
| yellow | LRRC49 |
| yellow | LRRC4 |
| blue | LRRC59 |
| blue | LRRC8A |
| blue | LRRC8E |
| yellow | LSAMP |
| green | LSM4 |
| green | LSM7 |
| blue | LSP1 |
| green | LSR |
| pink | LST1 |
| grey | LTB4R2 |
| brown | LTBP2 |
| brown | LTBP3 |
| grey | LTBP4 |
| blue | LTBR |
| blue | LTB |
| yellow | LTF |
| grey | LTV1 |
| red | LUC7L3 |
| red | LUC7L |
| brown | LUM |
| grey | LXN |
| blue | LY86 |
| pink | LY96 |
| blue | LY9 |
| blue | LYL1 |
| brown | LYPD1 |
| magenta | LYPD3 |
| blue | LYPD6B |
| blue | LYPLA1 |
| magenta | LYPLA2P1 |
| green | LYRM1 |
| grey | LYRM2 |
| brown | LYRM5 |
| blue | LYSMD1 |
| pink | LYZ |
| yellow | LZTS1 |
| magenta | MACC1 |
| brown | MAD2L1 |
| magenta | MADD |
| blue | MAFF |
| brown | MAFG |
| blue | MAFK |
| yellow | MAF |
| blue | MAGED1 |
| brown | MAGED4B |
| green | MAGED4 |
| grey | MAGEE1 |
| brown | MAGEH1 |
| grey | MAL2 |
| red | MALAT1 |
| magenta | MALL |
| blue | MALT1 |
| red | MAMDC4 |
| yellow | MAMLD1 |
| blue | MAN1C1 |
| blue | MAN2A2 |
| blue | MAN2B1 |
| pink | MANBA |
| grey | MANEAL |
| green | MAOB |
| brown | MAP1A |
| brown | MAP1B |
| green | MAP1S |
| green | MAP2K2 |
| green | MAP2K5 |
| green | MAP2K7 |
| brown | MAP2 |
| magenta | MAP3K12 |
| yellow | MAP3K14 |
| blue | MAP4K1 |
| yellow | MAP7D2 |
| green | MAP7D3 |
| green | MAP9 |
| magenta | MAPK13 |
| grey | MAPK7 |
| green | MAPK8IP2 |
| red | MAPK8IP3 |
| grey | MAPK9 |
| green | MAPKAPK5 |
| green | MAPKBP1 |
| green | MAPKSP1 |
| brown | MAPRE3 |
| blue | 1-Mar |
| yellow | 3-Mar |
| pink | MARCO |
| brown | MARK1 |
| brown | MARK4 |
| green | MARS |
| brown | MARVELD1 |
| blue | MAST3 |
| pink | MASTL |
| brown | MAT2A |
| yellow | MAT2B |
| blue | MATK |
| yellow | MATN2 |
| brown | MAVS |
| blue | MAX |
| green | MAZ |
| brown | MBD1 |
| green | MBD3 |
| yellow | MBNL2 |
| grey | MBOAT1 |
| magenta | MBOAT2 |
| brown | MCAM |
| yellow | MCF2L |
| brown | MCM3 |
| brown | MCM5 |
| green | MCM7 |
| green | MCOLN1 |
| blue | MCOLN2 |
| brown | ME3 |
| grey | MEAF6 |
| green | MED16 |
| pink | MED24 |
| green | MED25 |
| red | MED26 |
| grey | MED29 |
| green | MED30 |
| brown | MED6 |
| blue | MEF2B |
| yellow | MEGF10 |
| yellow | MEGF6 |
| yellow | MEGF8 |
| red | MEI1 |
| brown | MEIS1 |
| green | MEIS2 |
| brown | MELK |
| brown | MEN1 |
| blue | MEOX1 |
| yellow | MERTK |
| grey | MESDC2 |
| red | METT11D1 |
| red | METTL10 |
| yellow | METTL13 |
| blue | METTL2A |
| red | METTL3 |
| blue | METTL7A |
| grey | METTL9 |
| blue | MEX3D |
| brown | MFAP2 |
| brown | MFAP4 |
| brown | MFAP5 |
| brown | MFGE8 |
| blue | MFNG |
| brown | MFRP |
| yellow | MFSD2A |
| pink | MFSD7 |
| green | MFSD8 |
| yellow | MGAT3 |
| blue | MGAT4A |
| yellow | MGC2752 |
| blue | MGC29506 |
| pink | MGC57346 |
| brown | MGP |
| blue | MIAT |
| blue | MICAL1 |
| brown | MICAL2 |
| green | MICAL3 |
| magenta | MICALL1 |
| yellow | MICALL2 |
| grey | MID1IP1 |
| green | MID1 |
| green | MIER2 |
| yellow | MINA |
| grey | MINPP1 |
| green | MIOS |
| red | MITD1 |
| yellow | MKL1 |
| grey | MKNK1 |
| pink | MKS1 |
| brown | MLF1IP |
| brown | MLF1 |
| brown | MLLT11 |
| green | MLLT1 |
| magenta | MLLT3 |
| blue | MLLT6 |
| grey | MLPH |
| green | MMAA |
| blue | MMADHC |
| yellow | MMD |
| brown | MME |
| blue | MMP10 |
| brown | MMP11 |
| pink | MMP12 |
| brown | MMP13 |
| brown | MMP14 |
| blue | MMP15 |
| yellow | MMP19 |
| brown | MMP1 |
| pink | MMP25 |
| yellow | MMP28 |
| brown | MMP2 |
| brown | MMP3 |
| pink | MMP9 |
| brown | MMRN2 |
| brown | MN1 |
| pink | MNDA |
| brown | MNS1 |
| blue | MOBKL2A |
| yellow | MOBKL2B |
| brown | MOBKL2C |
| green | MOBKL3 |
| brown | MOCS1 |
| blue | MORC2 |
| blue | MORF4L2 |
| yellow | MOXD1 |
| pink | MPEG1 |
| grey | MPHOSPH10 |
| yellow | MPI |
| green | MPND |
| pink | MPP1 |
| red | MPP3 |
| yellow | MPP6 |
| red | MPPE1 |
| green | MPRIP |
| green | MPV17L2 |
| brown | MPZL1 |
| magenta | MPZL2 |
| green | MR1 |
| brown | MRAS |
| pink | MRC1 |
| brown | MRC2 |
| brown | MRGPRF |
| red | MRI1 |
| green | MRPL10 |
| green | MRPL11 |
| green | MRPL13 |
| green | MRPL15 |
| green | MRPL34 |
| brown | MRPL35 |
| brown | MRPL39 |
| blue | MRPL44 |
| blue | MRPL49 |
| green | MRPL4 |
| brown | MRPL50 |
| green | MRPL54 |
| green | MRPS12 |
| green | MRPS30 |
| green | MRPS35 |
| brown | MRRF |
| brown | MRVI1 |
| blue | MS4A1 |
| pink | MS4A4A |
| pink | MS4A6A |
| pink | MS4A7 |
| brown | MSC |
| red | MSH5 |
| grey | MSL3L2 |
| blue | MSL3 |
| green | MSLN |
| yellow | MSMB |
| pink | MSR1 |
| brown | MSRB3 |
| red | MST1P2 |
| yellow | MST1R |
| yellow | MSTO1 |
| green | MT1G |
| brown | MTA2 |
| blue | MTA3 |
| green | MTERFD1 |
| blue | MTERFD2 |
| red | MTERFD3 |
| yellow | MTHFD1L |
| brown | MTHFD2 |
| grey | MTIF2 |
| pink | MTL5 |
| green | MTMR11 |
| blue | MTSS1L |
| brown | MTX2 |
| yellow | MUC15 |
| magenta | MUC20 |
| green | MUM1 |
| red | MUS81 |
| magenta | MXD1 |
| blue | MXD4 |
| brown | MXRA5 |
| brown | MXRA7 |
| brown | MXRA8 |
| brown | MYADM |
| green | MYBBP1A |
| yellow | MYBL1 |
| grey | MYB |
| magenta | MYCBP |
| grey | MYCL1 |
| yellow | MYCN |
| grey | MYC |
| pink | MYEOV |
| brown | MYH11 |
| blue | MYH14 |
| red | MYH9 |
| red | MYL5 |
| brown | MYL9 |
| brown | MYLK |
| red | MYO15B |
| brown | MYO19 |
| blue | MYO1F |
| blue | MYO1G |
| grey | MYO3A |
| pink | MYO7A |
| blue | MYO9B |
| red | MZF1 |
| blue | N4BP2L1 |
| blue | N4BP2L2 |
| blue | NAAA |
| yellow | NACC1 |
| pink | NADK |
| red | NAPB |
| blue | NAPSB |
| red | NASP |
| grey | NAT1 |
| yellow | NAV2 |
| magenta | NBEAL2 |
| green | NBEA |
| yellow | NCALD |
| brown | NCAPG |
| grey | NCDN |
| blue | NCF1C |
| blue | NCF1 |
| pink | NCF2 |
| blue | NCF4 |
| blue | NCKAP1L |
| brown | NCKAP5L |
| green | NCKAP5 |
| green | NCLN |
| green | NCRNA00174 |
| red | NCRNA00201 |
| blue | NCS1 |
| brown | NDC80 |
| grey | NDNL2 |
| brown | NDN |
| grey | NDRG1 |
| magenta | NDRG2 |
| grey | NDRG4 |
| blue | NDST2 |
| green | NDUFA11 |
| green | NDUFA13 |
| grey | NDUFA4L2 |
| green | NDUFA7 |
| green | NDUFAB1 |
| green | NDUFB7 |
| green | NDUFS7 |
| red | NEAT1 |
| green | NECAB1 |
| blue | NECAP2 |
| yellow | NEDD1 |
| blue | NEDD4L |
| brown | NEFH |
| grey | NEFL |
| grey | NEIL2 |
| brown | NEK11 |
| pink | NEK6 |
| blue | NEK8 |
| grey | NELL2 |
| red | NEURL4 |
| brown | NF2 |
| pink | NFAM1 |
| grey | NFATC1 |
| brown | NFATC4 |
| yellow | NFE2L3 |
| green | NFIA |
| magenta | NFIB |
| yellow | NFIL3 |
| green | NFKB1 |
| yellow | NFKB2 |
| yellow | NFKBIA |
| blue | NFKBID |
| yellow | NFKBIE |
| brown | NFS1 |
| red | NFYB |
| blue | NGEF |
| yellow | NGFR |
| grey | NHEJ1 |
| blue | NHLRC3 |
| brown | NID1 |
| brown | NID2 |
| brown | NIF3L1 |
| yellow | NINJ1 |
| pink | NINJ2 |
| red | NINL |
| blue | NIPSNAP1 |
| blue | NKG7 |
| blue | NKIRAS2 |
| grey | NKX3.1 |
| grey | NLGN4Y |
| green | NLK |
| blue | NLRC3 |
| yellow | NLRP1 |
| pink | NLRP2 |
| brown | NMNAT1 |
| yellow | NMT2 |
| brown | NNMT |
| pink | NOD1 |
| red | NOMO1 |
| red | NOMO3 |
| grey | NOP14 |
| grey | NOP2 |
| brown | NOS2 |
| brown | NOS3 |
| yellow | NOTCH4 |
| yellow | NOV |
| brown | NOX4 |
| yellow | NOXO1 |
| brown | NPAS2 |
| red | NPIPL3 |
| red | NPIP |
| blue | NPLOC4 |
| pink | NPL |
| brown | NPM2 |
| yellow | NPNT |
| brown | NPR1 |
| yellow | NPTXR |
| blue | NR1D1 |
| green | NR1H2 |
| pink | NR1H3 |
| green | NR2C2AP |
| brown | NR2F1 |
| green | NR2F6 |
| brown | NR4A3 |
| blue | NRARP |
| yellow | NRCAM |
| blue | NRIP3 |
| brown | NRP1 |
| red | NSMCE4A |
| blue | NSUN2 |
| red | NSUN5P1 |
| red | NSUN5P2 |
| brown | NSUN6 |
| brown | NSUN7 |
| yellow | NT5DC1 |
| magenta | NT5DC3 |
| brown | NT5E |
| brown | NTM |
| yellow | NTN1 |
| brown | NTN4 |
| yellow | NTRK2 |
| yellow | NTS |
| yellow | NUAK1 |
| green | NUAK2 |
| blue | NUB1 |
| green | NUBP1 |
| grey | NUBPL |
| brown | NUDCD3 |
| magenta | NUDT11 |
| blue | NUDT12 |
| grey | NUDT15 |
| grey | NUDT19 |
| brown | NUF2 |
| grey | NUFIP1 |
| blue | NUMBL |
| brown | NUP210 |
| brown | NUP35 |
| yellow | NUP50 |
| green | NUP54 |
| red | NUPL2 |
| brown | NUSAP1 |
| red | NVL |
| red | NXF1 |
| yellow | NXN |
| green | NXPH4 |
| pink | NYNRIN |
| brown | OAF |
| green | OAZ1 |
| brown | OAZ2 |
| grey | OBFC2A |
| brown | OBSL1 |
| yellow | OCA2 |
| yellow | ODC1 |
| magenta | ODF2L |
| grey | ODF2 |
| green | ODZ2 |
| yellow | ODZ3 |
| brown | ODZ4 |
| red | OFD1 |
| red | OGFOD2 |
| blue | OGFRL1 |
| brown | OIP5 |
| yellow | OLFM1 |
| brown | OLFM2 |
| brown | OLFML1 |
| green | OLFML2A |
| brown | OLFML2B |
| brown | OLFML3 |
| pink | OLR1 |
| grey | OMA1 |
| yellow | ORAI2 |
| blue | ORAI3 |
| pink | ORC4L |
| green | ORC5L |
| brown | ORC6L |
| red | ORMDL1 |
| blue | OSBP2 |
| brown | OSBPL5 |
| red | OSBPL7 |
| pink | OSCAR |
| yellow | OSTF1 |
| brown | OSTM1 |
| green | OSTalpha |
| magenta | OTUB2 |
| blue | OTUD1 |
| magenta | OVOL1 |
| green | OVOL2 |
| yellow | OXCT1 |
| blue | P2RX5 |
| blue | P2RY10 |
| green | P2RY11 |
| blue | P2RY13 |
| magenta | P2RY2 |
| pink | P2RY6 |
| blue | P2RY8 |
| grey | P4HA1 |
| blue | P4HA2 |
| green | PA2G4P4 |
| green | PA2G4 |
| red | PABPC1L |
| green | PABPC4L |
| red | PABPN1 |
| grey | PACS1 |
| magenta | PADI1 |
| grey | PADI3 |
| green | PAFAH1B3 |
| magenta | PAFAH2 |
| blue | PAG1 |
| blue | PAIP1 |
| blue | PAIP2B |
| grey | PAK1IP1 |
| yellow | PAK1 |
| blue | PAK4 |
| yellow | PAK6 |
| brown | PALM2.AKAP2 |
| green | PALMD |
| brown | PALM |
| yellow | PAMR1 |
| red | PANX1 |
| yellow | PAPLN |
| green | PAPPA |
| yellow | PAPSS1 |
| yellow | PAPSS2 |
| brown | PAQR4 |
| grey | PAQR5 |
| blue | PAQR7 |
| blue | PAQR8 |
| blue | PARD3 |
| grey | PARD6B |
| grey | PARD6G |
| yellow | PARM1 |
| red | PARP10 |
| blue | PARP11 |
| brown | PARP16 |
| brown | PARP2 |
| red | PARP6 |
| brown | PARS2 |
| grey | PART1 |
| blue | PARVG |
| grey | PAX1 |
| blue | PAX5 |
| grey | PAX6 |
| grey | PAX8 |
| brown | PAX9 |
| brown | PBK |
| brown | PBXIP1 |
| brown | PCBD2 |
| grey | PCBP1 |
| yellow | PCCA |
| brown | PCDH12 |
| brown | PCDH17 |
| brown | PCDH18 |
| green | PCDH1 |
| red | PCDH7 |
| blue | PCDHB14 |
| red | PCDHGC3 |
| blue | PCGF2 |
| grey | PCGF3 |
| grey | PCGF6 |
| grey | PCID2 |
| grey | PCNT |
| blue | PCNXL3 |
| grey | PCOLCE2 |
| brown | PCOLCE |
| grey | PCP4L1 |
| brown | PCSK5 |
| blue | PCSK7 |
| grey | PCTP |
| brown | PCYOX1L |
| pink | PDCD1LG2 |
| blue | PDCD1 |
| grey | PDCD7 |
| red | PDDC1 |
| green | PDE10A |
| brown | PDE2A |
| brown | PDE4A |
| brown | PDE4B |
| grey | PDE7A |
| grey | PDE9A |
| brown | PDGFA |
| brown | PDGFB |
| brown | PDGFC |
| brown | PDGFRA |
| brown | PDGFRB |
| brown | PDGFRL |
| red | PDIA4 |
| blue | PDIA5 |
| red | PDIA6 |
| grey | PDK2 |
| grey | PDK3 |
| yellow | PDPN |
| brown | PDSS1 |
| red | PDXDC2 |
| yellow | PDZD2 |
| magenta | PDZK1IP1 |
| brown | PDZRN3 |
| brown | PEA15 |
| pink | PECAM1 |
| brown | PECR |
| brown | PEG10 |
| green | PELI2 |
| magenta | PERP |
| blue | PEX11A |
| brown | PEX5 |
| brown | PEX6 |
| grey | PEX7 |
| yellow | PFKFB4 |
| blue | PFKP |
| grey | PFN2 |
| brown | PGAP2 |
| grey | PGBD1 |
| brown | PGBD2 |
| green | PGBD3 |
| brown | PGCP |
| magenta | PGD |
| green | PGLS |
| magenta | PGLYRP3 |
| blue | PGM1 |
| red | PGM2L1 |
| blue | PGPEP1 |
| green | PGRMC2 |
| red | PGS1 |
| green | PHB |
| yellow | PHC1 |
| yellow | PHF10 |
| blue | PHF13 |
| grey | PHF15 |
| blue | PHF17 |
| red | PHKA2 |
| blue | PHLDA1 |
| blue | PHLDA2 |
| brown | PHLDB1 |
| grey | PHYHD1 |
| red | PI4KAP1 |
| red | PI4KAP2 |
| grey | PI4KB |
| green | PIAS3 |
| red | PIF1 |
| grey | PIGG |
| grey | PIGR |
| blue | PIK3AP1 |
| green | PIK3C2B |
| blue | PIK3CD |
| blue | PIK3CG |
| blue | PIK3IP1 |
| grey | PIK3R2 |
| blue | PIK3R5 |
| pink | PILRA |
| red | PILRB |
| magenta | PIM1 |
| blue | PIM2 |
| green | PIN1 |
| blue | PION |
| blue | PIP4K2A |
| brown | PIR |
| yellow | PISD |
| blue | PITPNB |
| blue | PITPNC1 |
| grey | PITPNM1 |
| red | PITRM1 |
| green | PITX1 |
| brown | PITX2 |
| yellow | PJA1 |
| green | PKD2 |
| yellow | PKDCC |
| brown | PKIG |
| grey | PKNOX1 |
| magenta | PKP1 |
| yellow | PKP2 |
| blue | PKP3 |
| green | PKP4 |
| blue | PLA2G12A |
| blue | PLA2G2D |
| brown | PLA2G3 |
| yellow | PLA2G4C |
| magenta | PLA2G4F |
| red | PLA2G6 |
| pink | PLA2G7 |
| magenta | PLAC2 |
| grey | PLAC8 |
| red | PLAU |
| magenta | PLBD1 |
| red | PLBD2 |
| blue | PLCB2 |
| blue | PLCB3 |
| blue | PLCD3 |
| blue | PLCG2 |
| brown | PLCH2 |
| yellow | PLCL1 |
| blue | PLCL2 |
| red | PLCXD1 |
| pink | PLD3 |
| blue | PLD4 |
| yellow | PLD6 |
| red | PLEC |
| blue | PLEKHA2 |
| green | PLEKHA3 |
| blue | PLEKHA6 |
| blue | PLEKHB1 |
| yellow | PLEKHF1 |
| brown | PLEKHG3 |
| grey | PLEKHG4B |
| brown | PLEKHG4 |
| green | PLEKHG5 |
| pink | PLEKHG6 |
| green | PLEKHH2 |
| green | PLEKHJ1 |
| magenta | PLEKHN1 |
| pink | PLEKHO1 |
| blue | PLEKHO2 |
| blue | PLEK |
| pink | PLIN2 |
| magenta | PLIN3 |
| yellow | PLK1 |
| grey | PLK2 |
| green | PLLP |
| brown | PLOD1 |
| green | PLRG1 |
| pink | PLTP |
| brown | PLVAP |
| brown | PLXDC1 |
| brown | PLXDC2 |
| blue | PLXNB2 |
| brown | PLXND1 |
| brown | PMEPA1 |
| brown | PMP22 |
| brown | PMS2L11 |
| brown | PNCK |
| brown | PNLDC1 |
| yellow | PNMAL2 |
| blue | PNO1 |
| green | PNPLA6 |
| yellow | PNPO |
| yellow | PNRC1 |
| blue | PNRC2 |
| brown | POC5 |
| brown | PODNL1 |
| brown | PODN |
| yellow | PODXL |
| brown | POLA2 |
| grey | POLDIP3 |
| brown | POLE3 |
| red | POLG2 |
| grey | POLG |
| blue | POLM |
| green | POLR2E |
| grey | POLR2J2 |
| green | POLR3D |
| yellow | POLR3G |
| green | POLR3K |
| green | POLRMT |
| green | PON2 |
| grey | PON3 |
| grey | POP1 |
| green | POP7 |
| blue | POR |
| brown | POSTN |
| blue | POU2AF1 |
| grey | POU2F1 |
| blue | POU2F2 |
| blue | POU6F1 |
| green | PPAN |
| brown | PPAP2A |
| yellow | PPAP2B |
| blue | PPAP2C |
| green | PPARGC1A |
| brown | PPARG |
| yellow | PPFIBP2 |
| brown | PPHLN1 |
| brown | PPIL3 |
| magenta | PPL |
| grey | PPM1F |
| blue | PPM1K |
| blue | PPM1M |
| blue | PPME1 |
| blue | PPP1CB |
| grey | PPP1R10 |
| magenta | PPP1R11 |
| brown | PPP1R13B |
| magenta | PPP1R13L |
| grey | PPP1R14C |
| blue | PPP1R16B |
| brown | PPP1R3C |
| red | PPP1R3E |
| blue | PPP1R9B |
| brown | PPP2CA |
| grey | PPP2CB |
| blue | PPP2R2B |
| yellow | PPP2R3A |
| yellow | PPP2R5A |
| blue | PPP2R5B |
| green | PPP3CA |
| brown | PPP3CB |
| green | PPP3CC |
| brown | PPP4R1 |
| yellow | PPP4R4 |
| pink | PPT1 |
| pink | PQLC3 |
| grey | PRAME |
| brown | PRCP |
| green | PRDX2 |
| brown | PRELP |
| blue | PREX1 |
| blue | PRF1 |
| brown | PRICKLE1 |
| brown | PRICKLE2 |
| magenta | PRICKLE4 |
| blue | PRIMA1 |
| yellow | PRKAB1 |
| yellow | PRKAG2 |
| blue | PRKAR2B |
| blue | PRKCB |
| pink | PRKCQ |
| green | PRKCSH |
| yellow | PRKD1 |
| grey | PRKY |
| yellow | PRLR |
| green | PRMT10 |
| grey | PRNP |
| grey | PROCR |
| yellow | PRODH |
| magenta | PROM2 |
| yellow | PROS1 |
| magenta | PROSC |
| green | PRPF19 |
| brown | PRPF38A |
| pink | PRPSAP1 |
| blue | PRR15 |
| yellow | PRR5L |
| yellow | PRRX1 |
| brown | PRSS16 |
| blue | PRSS21 |
| magenta | PRSS22 |
| brown | PRSS23 |
| magenta | PRSS8 |
| blue | PRTFDC1 |
| pink | PSAP |
| brown | PSAT1 |
| blue | PSD4 |
| red | PSMC3IP |
| blue | PSMD14 |
| brown | PSMD1 |
| brown | PSPC1 |
| blue | PSTPIP1 |
| yellow | PSTPIP2 |
| red | PTBP2 |
| yellow | PTCD1 |
| red | PTCD3 |
| brown | PTENP1 |
| brown | PTEN |
| blue | PTGDS |
| blue | PTGER4 |
| green | PTGES3 |
| yellow | PTGES |
| grey | PTGR2 |
| brown | PTGS1 |
| blue | PTK2B |
| magenta | PTK6 |
| brown | PTK7 |
| yellow | PTN |
| brown | PTP4A3 |
| green | PTPN12 |
| blue | PTPN22 |
| green | PTPN3 |
| blue | PTPN6 |
| blue | PTPN7 |
| green | PTPRA |
| blue | PTPRCAP |
| blue | PTPRC |
| magenta | PTPRH |
| blue | PTPRJ |
| yellow | PTPRM |
| blue | PTPRN2 |
| yellow | PTPRS |
| blue | PTPRU |
| brown | PTRF |
| grey | PTTG1IP |
| green | PUS10 |
| blue | PVRIG |
| magenta | PVRL4 |
| blue | PVR |
| green | PWP2 |
| brown | PXDN |
| red | PXN |
| yellow | PYCARD |
| blue | PYCR1 |
| yellow | PYGL |
| blue | PYHIN1 |
| blue | ProSAPiP1 |
| yellow | QDPR |
| pink | QPCT |
| red | QRICH2 |
| grey | QRSL1 |
| red | QSOX1 |
| red | QSOX2 |
| red | QTRT1 |
| magenta | RAB10 |
| magenta | RAB11A |
| green | RAB11B |
| brown | RAB11FIP3 |
| yellow | RAB11FIP4 |
| brown | RAB11FIP5 |
| grey | RAB12 |
| blue | RAB1A |
| pink | RAB20 |
| magenta | RAB25 |
| red | RAB28 |
| red | RAB2A |
| yellow | RAB35 |
| yellow | RAB36 |
| blue | RAB37 |
| brown | RAB3D |
| brown | RAB3IL1 |
| grey | RAB3IP |
| blue | RAB40B |
| grey | RAB40C |
| yellow | RAB42 |
| red | RAB5C |
| grey | RAB7L1 |
| blue | RAB8A |
| blue | RAB8B |
| yellow | RAB9A |
| blue | RABGEF1 |
| red | RABL2A |
| green | RABL2B |
| blue | RAC2 |
| grey | RAD1 |
| green | RAD23A |
| green | RAD51C |
| magenta | RAD51L3 |
| brown | RAD54L |
| red | RAD9A |
| brown | RAD9B |
| magenta | RAET1E |
| magenta | RAET1G |
| blue | RAET1L |
| grey | RAF1 |
| yellow | RAI14 |
| blue | RAI2 |
| magenta | RALA |
| yellow | RALB |
| red | RALGPS1 |
| yellow | RAMP1 |
| blue | RAMP3 |
| green | RANBP3 |
| yellow | RAP2B |
| blue | RAPGEF1 |
| magenta | RAPGEF3 |
| magenta | RAPGEFL1 |
| brown | RARA |
| magenta | RARG |
| pink | RARRES1 |
| brown | RARRES2 |
| grey | RARS2 |
| brown | RASA3 |
| blue | RASA4P |
| brown | RASA4 |
| magenta | RASAL1 |
| blue | RASAL3 |
| brown | RASD1 |
| yellow | RASD2 |
| yellow | RASGEF1A |
| yellow | RASGEF1B |
| blue | RASGRP1 |
| blue | RASGRP2 |
| blue | RASGRP3 |
| brown | RASL12 |
| grey | RASSF10 |
| blue | RASSF2 |
| pink | RASSF4 |
| blue | RASSF5 |
| green | RAVER1 |
| brown | RBBP7 |
| brown | RBM14 |
| yellow | RBM19 |
| grey | RBM23 |
| yellow | RBM38 |
| brown | RBM39 |
| green | RBM42 |
| brown | RBM45 |
| red | RBM5 |
| red | RBM6 |
| green | RBMS1 |
| red | RBMX |
| blue | RBP1 |
| green | RBP7 |
| magenta | RBPJ |
| brown | RBPMS |
| yellow | RCAN1 |
| brown | RCAN2 |
| brown | RCC1 |
| brown | RCC2 |
| green | RCHY1 |
| green | RCL1 |
| grey | RCN2 |
| brown | RCN3 |
| green | RCOR3 |
| blue | RCSD1 |
| green | RDH13 |
| yellow | RDH16 |
| brown | RECK |
| red | RECQL5 |
| green | REEP4 |
| magenta | REEP6 |
| grey | RELA |
| yellow | RELB |
| blue | RELT |
| pink | RENBP |
| green | REXO1 |
| grey | RFK |
| brown | RFPL1S |
| blue | RFTN1 |
| green | RFXANK |
| red | RG9MTD3 |
| yellow | RGAG4 |
| yellow | RGMA |
| blue | RGS10 |
| brown | RGS16 |
| blue | RGS19 |
| blue | RGS1 |
| brown | RGS2 |
| brown | RGS3 |
| brown | RGS4 |
| brown | RGS5 |
| magenta | RHBDL2 |
| magenta | RHCG |
| red | RHEBL1 |
| brown | RHOBTB1 |
| yellow | RHOBTB2 |
| blue | RHOB |
| blue | RHOF |
| blue | RHOH |
| brown | RHOQ |
| brown | RHOU |
| brown | RIBC2 |
| blue | RILPL2 |
| green | RIMS3 |
| blue | RIN3 |
| blue | RINL |
| grey | RIOK2 |
| green | RIPK1 |
| brown | RIPK4 |
| blue | RLTPR |
| pink | RNASE1 |
| pink | RNASE6 |
| blue | RNASEH1 |
| green | RNASEH2A |
| yellow | RND1 |
| magenta | RND3 |
| green | RNF103 |
| brown | RNF122 |
| blue | RNF125 |
| green | RNF126 |
| green | RNF130 |
| red | RNF139 |
| brown | RNF145 |
| yellow | RNF150 |
| red | RNF152 |
| blue | RNF157 |
| yellow | RNF165 |
| pink | RNF166 |
| grey | RNF185 |
| yellow | RNF19A |
| yellow | RNF19B |
| red | RNF207 |
| grey | RNF212 |
| blue | RNF213 |
| brown | RNF214 |
| yellow | RNF216 |
| brown | RNF24 |
| brown | RNF34 |
| blue | RNF39 |
| yellow | RNF44 |
| brown | RNF4 |
| grey | RNLS |
| yellow | ROBO1 |
| yellow | ROBO2 |
| brown | ROBO4 |
| yellow | ROR2 |
| yellow | RORB |
| red | RPAIN |
| yellow | RPH3AL |
| yellow | RPIA |
| green | RPS15 |
| grey | RPS28 |
| grey | RPS4Y1 |
| brown | RPS6KA2 |
| grey | RRAGD |
| grey | RRAS2 |
| yellow | RRAS |
| brown | RRM2 |
| grey | RRP15 |
| red | RRP7B |
| green | RRS1 |
| grey | RTCD1 |
| red | RTEL1 |
| pink | RTN1 |
| brown | RTN3 |
| yellow | RTN4RL1 |
| red | RUFY3 |
| brown | RUNX2 |
| blue | RUNX3 |
| blue | RUSC1 |
| brown | RUSC2 |
| green | RUVBL2 |
| blue | RXRA |
| yellow | RYR3 |
| magenta | S100A12 |
| magenta | S100A14 |
| magenta | S100A16 |
| pink | S100A4 |
| magenta | S100A8 |
| magenta | S100A9 |
| blue | S100B |
| brown | S1PR1 |
| blue | S1PR2 |
| blue | S1PR4 |
| green | SAE1 |
| green | SAFB2 |
| green | SAFB |
| grey | SALL2 |
| green | SAMD1 |
| brown | SAMD4A |
| blue | SAMD9L |
| blue | SAMSN1 |
| yellow | SAP30L |
| red | SAP30 |
| red | SAR1B |
| grey | SARM1 |
| blue | SASH3 |
| green | SAT1 |
| grey | SBDSP1 |
| blue | SBDS |
| magenta | SC4MOL |
| green | SC5DL |
| green | SCAF1 |
| magenta | SCAMP2 |
| green | SCAMP4 |
| green | SCAMP5 |
| red | SCAND2 |
| brown | SCARA3 |
| pink | SCARB1 |
| green | SCARB2 |
| pink | SCARF1 |
| brown | SCARF2 |
| brown | SCCPDH |
| yellow | SCD5 |
| grey | SCFD1 |
| grey | SCHIP1 |
| green | SCIN |
| green | SCLT1 |
| grey | SCMH1 |
| brown | SCML1 |
| magenta | SCNN1A |
| grey | SCNN1B |
| yellow | SCNN1G |
| green | SCO1 |
| green | SCOC |
| yellow | SCUBE2 |
| green | SCYL3 |
| magenta | SDC1 |
| brown | SDC2 |
| pink | SDC3 |
| grey | SDC4 |
| magenta | SDCBP2 |
| grey | SDCCAG8 |
| green | SDHA |
| yellow | SDK1 |
| yellow | SDK2 |
| brown | SDPR |
| grey | SDR16C5 |
| pink | SDS |
| blue | SEC14L2 |
| red | SEC31A |
| red | SEC31B |
| red | SECISBP2 |
| blue | SEL1L3 |
| grey | SELENBP1 |
| brown | SELE |
| blue | SELL |
| blue | SELPLG |
| blue | SELP |
| blue | SEMA3B |
| red | SEMA3C |
| magenta | SEMA3F |
| brown | SEMA3G |
| blue | SEMA4A |
| yellow | SEMA4C |
| blue | SEMA4D |
| yellow | SEMA5A |
| yellow | SEMA6A |
| brown | SEMA6B |
| brown | SEMA6D |
| blue | SEMA7A |
| pink | SEPHS1 |
| green | SEPHS2 |
| brown | SEPN1 |
| blue | 1-Sep |
| brown | 3-Sep |
| brown | 4-Sep |
| blue | 5-Sep |
| blue | 6-Sep |
| red | SEPT7P2 |
| green | 7-Sep |
| red | 9-Sep |
| grey | SERHL |
| pink | SERPINA1 |
| brown | SERPINA3 |
| magenta | SERPINB13 |
| magenta | SERPINB1 |
| magenta | SERPINB2 |
| magenta | SERPINB3 |
| magenta | SERPINB4 |
| magenta | SERPINB5 |
| brown | SERPINE1 |
| yellow | SERPINE2 |
| yellow | SERPINF1 |
| pink | SERPING1 |
| brown | SERPINH1 |
| green | SERTAD2 |
| yellow | SERTAD3 |
| blue | SESN1 |
| red | SETD4 |
| brown | SETD8 |
| blue | SETDB2 |
| yellow | SEZ6L2 |
| brown | SF1 |
| green | SF3A2 |
| blue | SF3B4 |
| green | SF4 |
| red | SFI1 |
| brown | SFPQ |
| brown | SFRP1 |
| brown | SFRP2 |
| brown | SFRP4 |
| red | SFRS16 |
| red | SFRS17A |
| grey | SFRS2B |
| red | SFRS2 |
| brown | SFRS4 |
| red | SFRS5 |
| red | SFRS6 |
| red | SFRS7 |
| red | SFRS8 |
| brown | SFT2D2 |
| grey | SFXN1 |
| grey | SFXN2 |
| brown | SFXN3 |
| brown | SGCB |
| brown | SGCD |
| brown | SGCE |
| grey | SGEF |
| blue | SGPP1 |
| red | SGSM2 |
| green | SGTA |
| blue | SGTB |
| red | SH2B1 |
| yellow | SH2B3 |
| blue | SH2D1A |
| blue | SH2D2A |
| green | SH2D3A |
| blue | SH2D3C |
| blue | SH3BGRL |
| brown | SH3BP1 |
| blue | SH3BP2 |
| magenta | SH3BP5L |
| yellow | SH3BP5 |
| green | SH3GL1 |
| blue | SH3KBP1 |
| brown | SH3RF3 |
| yellow | SH3TC1 |
| brown | SH3YL1 |
| green | SHANK2 |
| brown | SHANK3 |
| brown | SHC1 |
| brown | SHC2 |
| yellow | SHCBP1 |
| brown | SHE |
| yellow | SHISA2 |
| brown | SHMT1 |
| green | SHMT2 |
| yellow | SHOX2 |
| pink | SHROOM3 |
| brown | SHROOM4 |
| red | SIAE |
| blue | SIDT1 |
| blue | SIDT2 |
| pink | SIGLEC10 |
| pink | SIGLEC1 |
| green | SIGMAR1 |
| yellow | SIK1 |
| yellow | SIM2 |
| blue | SIPA1 |
| pink | SIRPA |
| pink | SIRPB1 |
| blue | SIRPG |
| green | SIRT6 |
| blue | SIT1 |
| blue | SIX1 |
| green | SIX2 |
| brown | SKA1 |
| brown | SKA2 |
| brown | SKA3 |
| blue | SKAP1 |
| blue | SLA2 |
| blue | SLAMF1 |
| blue | SLAMF6 |
| blue | SLAMF7 |
| pink | SLAMF8 |
| blue | SLA |
| brown | SLBP |
| pink | SLC11A1 |
| grey | SLC12A4 |
| yellow | SLC12A7 |
| yellow | SLC12A8 |
| magenta | SLC15A2 |
| pink | SLC15A3 |
| brown | SLC15A4 |
| grey | SLC16A14 |
| brown | SLC16A2 |
| blue | SLC16A5 |
| blue | SLC17A9 |
| blue | SLC19A2 |
| green | SLC1A1 |
| pink | SLC1A3 |
| green | SLC1A5 |
| pink | SLC20A1 |
| blue | SLC20A2 |
| magenta | SLC22A15 |
| brown | SLC24A3 |
| green | SLC25A10 |
| grey | SLC25A12 |
| blue | SLC25A13 |
| grey | SLC25A16 |
| green | SLC25A19 |
| yellow | SLC25A22 |
| brown | SLC25A23 |
| grey | SLC25A25 |
| red | SLC25A35 |
| brown | SLC25A37 |
| green | SLC25A3 |
| green | SLC25A42 |
| green | SLC25A43 |
| blue | SLC25A45 |
| yellow | SLC26A9 |
| blue | SLC27A2 |
| yellow | SLC27A4 |
| green | SLC27A5 |
| brown | SLC29A1 |
| brown | SLC29A2 |
| pink | SLC29A3 |
| pink | SLC2A3 |
| pink | SLC2A5 |
| pink | SLC2A6 |
| blue | SLC2A9 |
| pink | SLC31A2 |
| grey | SLC34A2 |
| blue | SLC35A2 |
| green | SLC35A4 |
| magenta | SLC35C1 |
| blue | SLC35E2 |
| yellow | SLC35F2 |
| grey | SLC37A1 |
| brown | SLC38A5 |
| pink | SLC38A6 |
| green | SLC39A11 |
| brown | SLC39A14 |
| magenta | SLC39A2 |
| green | SLC39A3 |
| green | SLC39A8 |
| brown | SLC41A2 |
| blue | SLC43A2 |
| grey | SLC44A2 |
| blue | SLC44A3 |
| grey | SLC44A4 |
| brown | SLC45A3 |
| yellow | SLC45A4 |
| pink | SLC46A3 |
| pink | SLC47A1 |
| grey | SLC4A2 |
| magenta | SLC6A14 |
| grey | SLC6A15 |
| yellow | SLC6A8 |
| yellow | SLC7A2 |
| yellow | SLC7A5 |
| pink | SLC7A7 |
| brown | SLC7A8 |
| pink | SLC8A1 |
| yellow | SLC9A2 |
| magenta | SLC9A3R1 |
| yellow | SLC9A9 |
| yellow | SLCO2A1 |
| pink | SLCO2B1 |
| grey | SLCO4A1 |
| grey | SLFN13 |
| yellow | SLIT3 |
| brown | SLMO2 |
| grey | SLU7 |
| green | SMAD1 |
| yellow | SMAD7 |
| magenta | SMAGP |
| blue | SMAP2 |
| yellow | SMARCA2 |
| grey | SMARCA4 |
| grey | SMARCAL1 |
| grey | SMARCD1 |
| brown | SMC1B |
| brown | SMNDC1 |
| brown | SMOC2 |
| grey | SMOX |
| brown | SMO |
| brown | SMPD4 |
| yellow | SMPDL3A |
| grey | SMPDL3B |
| blue | SMTN |
| green | SMU1 |
| brown | SMYD2 |
| brown | SNAI2 |
| red | SNAPC4 |
| brown | SNCAIP |
| brown | SND1 |
| brown | SNED1 |
| red | SNHG10 |
| red | SNHG12 |
| red | SNHG1 |
| green | SNN |
| green | SNRNP25 |
| grey | SNRNP27 |
| red | SNRNP70 |
| brown | SNTB1 |
| brown | SNW1 |
| pink | SNX10 |
| blue | SNX20 |
| blue | SNX2 |
| magenta | SNX33 |
| grey | SNX3 |
| blue | SOCS2 |
| brown | SOD3 |
| green | SOLH |
| brown | SORBS2 |
| brown | SORCS2 |
| blue | SORD |
| yellow | SORL1 |
| grey | SORT1 |
| yellow | SOX15 |
| grey | SOX2 |
| green | SOX9 |
| blue | SP140L |
| blue | SP140 |
| grey | SPAG16 |
| green | SPAG1 |
| brown | SPARCL1 |
| brown | SPARC |
| grey | SPATA20 |
| green | SPATA5L1 |
| brown | SPC24 |
| brown | SPC25 |
| green | SPCS3 |
| grey | SPESP1 |
| green | SPG20 |
| pink | SPI1 |
| yellow | SPIB |
| green | SPIN3 |
| yellow | SPIRE1 |
| green | SPIRE2 |
| green | SPNS1 |
| blue | SPN |
| brown | SPOCK1 |
| blue | SPOCK2 |
| brown | SPON1 |
| brown | SPON2 |
| grey | SPOP |
| pink | SPP1 |
| green | SPPL2B |
| brown | SPRY4 |
| yellow | SPSB1 |
| blue | SPTBN2 |
| red | SPTBN5 |
| brown | SPTLC3 |
| yellow | SQSTM1 |
| magenta | SRD5A3 |
| brown | SREBF1 |
| red | SRGAP3 |
| pink | SRGN |
| blue | SRP68 |
| brown | SRPR |
| brown | SRPX2 |
| brown | SRPX |
| yellow | SRRM3 |
| red | SRRT |
| red | SS18L1 |
| brown | SS18 |
| brown | SSC5D |
| magenta | SSH3 |
| yellow | SSPN |
| blue | SSRP1 |
| brown | ST3GAL2 |
| blue | ST3GAL5 |
| brown | ST3GAL6 |
| grey | ST5 |
| blue | ST6GAL1 |
| brown | ST6GAL2 |
| grey | ST6GALNAC2 |
| blue | ST7L |
| blue | ST7 |
| yellow | ST8SIA1 |
| blue | ST8SIA4 |
| pink | STAB1 |
| red | STAG3L3 |
| brown | STAG3 |
| blue | STAMBPL1 |
| blue | STAMBP |
| green | STAP2 |
| brown | STARD13 |
| grey | STARD3NL |
| brown | STARD8 |
| yellow | STAR |
| blue | STAT4 |
| blue | STAT5A |
| brown | STAT5B |
| blue | STC2 |
| green | STEAP3 |
| blue | STIP1 |
| blue | STK10 |
| green | STK11 |
| blue | STK17A |
| blue | STK17B |
| red | STK36 |
| brown | STK40 |
| blue | STK4 |
| green | STOML2 |
| grey | STOM |
| yellow | STON1 |
| yellow | STON2 |
| grey | STOX1 |
| grey | STRA6 |
| brown | STRAP |
| green | STRN4 |
| brown | STT3A |
| green | STX10 |
| pink | STX11 |
| red | STX16 |
| green | STX2 |
| blue | STX3 |
| yellow | STXBP1 |
| green | STXBP2 |
| yellow | STXBP6 |
| brown | SULF1 |
| brown | SULF2 |
| red | SULT1A3 |
| yellow | SULT1E1 |
| magenta | SULT2B1 |
| magenta | SUMF1 |
| magenta | SUOX |
| grey | SUPT3H |
| green | SUPT5H |
| red | SUPT7L |
| brown | SUSD2 |
| blue | SUSD3 |
| grey | SUSD4 |
| brown | SUV39H1 |
| brown | SUV39H2 |
| red | SUV420H2 |
| red | SUZ12P |
| yellow | SV2B |
| red | SVIL |
| grey | SVIP |
| blue | SYAP1 |
| grey | SYBU |
| brown | SYCP2 |
| brown | SYDE1 |
| blue | SYK |
| brown | SYNE1 |
| yellow | SYNGR2 |
| brown | SYNGR3 |
| red | SYNPO |
| blue | SYT11 |
| grey | SYT17 |
| blue | SYT7 |
| yellow | SYTL3 |
| blue | SYTL4 |
| blue | SYVN1 |
| green | TACO1 |
| magenta | TACSTD2 |
| red | TAF1C |
| grey | TAF6 |
| grey | TAF7L |
| brown | TAF7 |
| blue | TAGAP |
| brown | TAGLN |
| green | TANK |
| blue | TAPBPL |
| grey | TAPT1 |
| brown | TARDBP |
| blue | TARS2 |
| yellow | TARSL2 |
| grey | TARS |
| green | TATDN1 |
| green | TBC1D10B |
| blue | TBC1D10C |
| brown | TBC1D16 |
| brown | TBC1D1 |
| red | TBC1D3B |
| red | TBC1D3 |
| green | TBCK |
| yellow | TBKBP1 |
| yellow | TBL1X |
| green | TBL2 |
| grey | TBPL1 |
| grey | TBP |
| pink | TBRG1 |
| brown | TBX15 |
| brown | TBX1 |
| brown | TBX2 |
| grey | TBX3 |
| magenta | TBX6 |
| pink | TBXAS1 |
| green | TC2N |
| brown | TCAM1P |
| green | TCEA1 |
| brown | TCF19 |
| green | TCF3 |
| brown | TCF4 |
| yellow | TCF7L1 |
| green | TCF7L2 |
| blue | TCF7 |
| green | TCFL5 |
| red | TCHP |
| blue | TCL1A |
| magenta | TCN1 |
| pink | TCN2 |
| brown | TCOF1 |
| blue | TCP11 |
| grey | TCP1 |
| yellow | TCTN1 |
| grey | TCTN3 |
| yellow | TDRD10 |
| grey | TDRD5 |
| grey | TDRKH |
| magenta | TEAD3 |
| green | TECR |
| grey | TEF |
| brown | TEK |
| brown | TENC1 |
| yellow | TESC |
| green | TFAP2A |
| yellow | TFAP2C |
| green | TFAP4 |
| brown | TFB1M |
| blue | TFB2M |
| magenta | TFCP2L1 |
| pink | TFEC |
| blue | TFG |
| brown | TFPI |
| green | TGDS |
| green | TGFA |
| brown | TGFB1I1 |
| yellow | TGFB2 |
| brown | TGFB3 |
| brown | TGFBI |
| brown | TGFBR2 |
| blue | TGIF1 |
| yellow | TGIF2 |
| brown | TGM2 |
| yellow | TG |
| brown | THADA |
| brown | THAP10 |
| green | THAP2 |
| green | THAP6 |
| yellow | THAP8 |
| grey | THBD |
| brown | THBS1 |
| brown | THBS2 |
| brown | THBS3 |
| blue | THEM4 |
| blue | THEMIS |
| grey | THNSL2 |
| red | THOC1 |
| brown | THOC3 |
| green | THOP1 |
| blue | THRB |
| red | THSD1 |
| green | THSD4 |
| red | THUMPD2 |
| grey | THUMPD3 |
| brown | THY1 |
| brown | TIAL1 |
| brown | TIAM2 |
| magenta | TICAM1 |
| brown | TIE1 |
| brown | TIFA |
| red | TIGD1 |
| green | TIGD2 |
| grey | TIGD6 |
| blue | TIGIT |
| green | TIMM13 |
| green | TIMM44 |
| green | TIMM50 |
| brown | TIMP1 |
| brown | TIMP2 |
| brown | TIMP3 |
| yellow | TINAGL1 |
| brown | TIPIN |
| grey | TJP3 |
| green | TK1 |
| pink | TLE1 |
| grey | TLE2 |
| blue | TLK2 |
| red | TLN1 |
| brown | TLN2 |
| blue | TLR10 |
| yellow | TLR1 |
| pink | TLR4 |
| yellow | TLR6 |
| blue | TLR7 |
| pink | TLR8 |
| brown | TLX3 |
| magenta | TM4SF1 |
| pink | TM6SF1 |
| yellow | TM7SF3 |
| grey | TM9SF1 |
| blue | TMBIM1 |
| magenta | TMC4 |
| blue | TMC8 |
| yellow | TMCC3 |
| blue | TMCO1 |
| brown | TMCO4 |
| red | TMCO6 |
| green | TMED1 |
| grey | TMEM104 |
| blue | TMEM106A |
| green | TMEM109 |
| yellow | TMEM117 |
| brown | TMEM119 |
| blue | TMEM140 |
| green | TMEM143 |
| blue | TMEM149 |
| blue | TMEM14A |
| yellow | TMEM150C |
| magenta | TMEM154 |
| magenta | TMEM159 |
| green | TMEM160 |
| green | TMEM161A |
| magenta | TMEM165 |
| brown | TMEM170B |
| brown | TMEM171 |
| yellow | TMEM173 |
| pink | TMEM176A |
| pink | TMEM176B |
| green | TMEM180 |
| magenta | TMEM184A |
| red | TMEM184B |
| brown | TMEM200A |
| brown | TMEM201 |
| brown | TMEM204 |
| yellow | TMEM20 |
| brown | TMEM214 |
| grey | TMEM220 |
| blue | TMEM229B |
| grey | TMEM22 |
| magenta | TMEM231 |
| brown | TMEM2 |
| magenta | TMEM40 |
| brown | TMEM41B |
| grey | TMEM45A |
| brown | TMEM47 |
| brown | TMEM55A |
| grey | TMEM56 |
| grey | TMEM57 |
| grey | TMEM5 |
| green | TMEM63A |
| blue | TMEM66 |
| grey | TMEM68 |
| brown | TMEM69 |
| magenta | TMEM79 |
| pink | TMEM86A |
| brown | TMEM98 |
| green | TMEM99 |
| magenta | TMPRSS11A |
| magenta | TMPRSS11D |
| grey | TMPRSS2 |
| blue | TMPRSS4 |
| pink | TMSB15A |
| brown | TMTC1 |
| red | TMUB2 |
| brown | TMX4 |
| brown | TNC |
| yellow | TNFAIP2 |
| green | TNFAIP3 |
| brown | TNFAIP6 |
| blue | TNFAIP8L2 |
| blue | TNFAIP8 |
| green | TNFRSF10D |
| blue | TNFRSF12A |
| blue | TNFRSF14 |
| blue | TNFRSF17 |
| yellow | TNFRSF19 |
| blue | TNFRSF1A |
| blue | TNFRSF1B |
| red | TNFRSF25 |
| blue | TNFRSF4 |
| yellow | TNFRSF6B |
| yellow | TNFRSF9 |
| pink | TNFSF12.TNFSF13 |
| pink | TNFSF12 |
| blue | TNFSF13B |
| pink | TNFSF13 |
| magenta | TNFSF4 |
| green | TNFSF9 |
| blue | TNF |
| yellow | TNIP1 |
| brown | TNK1 |
| blue | TNKS1BP1 |
| yellow | TNS3 |
| blue | TNS4 |
| grey | TOMM20 |
| green | TOMM40 |
| blue | TOMM70A |
| red | TOP3B |
| blue | TOX2 |
| blue | TOX |
| blue | TP53AIP1 |
| yellow | TP53I11 |
| blue | TP53I3 |
| blue | TP53INP1 |
| blue | TP53INP2 |
| yellow | TP73 |
| blue | TPBG |
| brown | TPCN1 |
| grey | TPCN2 |
| yellow | TPD52L1 |
| brown | TPM1 |
| brown | TPM2 |
| grey | TPMT |
| pink | TPP1 |
| blue | TPPP3 |
| blue | TPPP |
| magenta | TPRXL |
| brown | TPSAB1 |
| brown | TPSB2 |
| blue | TPST1 |
| red | TRA2A |
| blue | TRAF1 |
| yellow | TRAF2 |
| blue | TRAF3IP3 |
| blue | TRAF5 |
| blue | TRAFD1 |
| blue | TRANK1 |
| green | TRAP1 |
| green | TRAPPC5 |
| brown | TRAPPC6B |
| grey | TRDMT1 |
| pink | TREM2 |
| brown | TRIM13 |
| blue | TRIM14 |
| yellow | TRIM16L |
| magenta | TRIM16 |
| green | TRIM28 |
| magenta | TRIM29 |
| blue | TRIM38 |
| grey | TRIM3 |
| grey | TRIM45 |
| yellow | TRIM47 |
| brown | TRIM59 |
| green | TRIM65 |
| green | TRIM68 |
| blue | TRIM7 |
| yellow | TRIM8 |
| blue | TRIP13 |
| magenta | TRIP4 |
| grey | TRMT12 |
| red | TRMT1 |
| magenta | TRNP1 |
| grey | TRNT1 |
| red | TROAP |
| brown | TRO |
| pink | TRPM2 |
| grey | TRPM4 |
| red | TRPV1 |
| pink | TRPV2 |
| brown | TRPV4 |
| yellow | TRPV6 |
| yellow | TSC22D1 |
| yellow | TSC2 |
| yellow | TSEN15 |
| grey | TSGA14 |
| blue | TSKU |
| grey | TSLP |
| green | TSNAX |
| blue | TSN |
| brown | TSPAN11 |
| brown | TSPAN12 |
| grey | TSPAN13 |
| yellow | TSPAN17 |
| brown | TSPAN18 |
| blue | TSPAN1 |
| blue | TSPAN33 |
| blue | TSPAN3 |
| pink | TSPAN4 |
| brown | TSPAN7 |
| yellow | TSPAN9 |
| red | TSPYL2 |
| brown | TSPYL5 |
| grey | TTC12 |
| magenta | TTC22 |
| grey | TTC23 |
| brown | TTC31 |
| blue | TTC39A |
| blue | TTC39C |
| grey | TTC7A |
| magenta | TTC9 |
| red | TTF1 |
| magenta | TTLL12 |
| red | TTLL3 |
| green | TTLL7 |
| grey | TTL |
| grey | TTTY15 |
| pink | TTYH2 |
| brown | TTYH3 |
| brown | TUBA1A |
| blue | TUBB2A |
| yellow | TUBB2B |
| brown | TUBD1 |
| grey | TUBE1 |
| blue | TUBG1 |
| red | TUBGCP6 |
| green | TUFM |
| yellow | TUSC3 |
| brown | TWIST1 |
| blue | TXNDC11 |
| blue | TXNDC5 |
| blue | TXNIP |
| blue | TXNRD3IT1 |
| yellow | TYK2 |
| red | TYMS |
| yellow | TYRO3 |
| pink | TYROBP |
| brown | U2AF2 |
| blue | UAP1 |
| red | UBA1 |
| blue | UBA7 |
| blue | UBASH3A |
| blue | UBASH3B |
| yellow | UBD |
| grey | UBE2D3 |
| brown | UBE2D4 |
| red | UBE2G2 |
| blue | UBE2J1 |
| grey | UBE2N |
| yellow | UBE2Q2 |
| yellow | UBE2QL1 |
| green | UBE2R2 |
| green | UBE2V2 |
| grey | UBIAD1 |
| green | UBL5 |
| grey | UBLCP1 |
| brown | UBTD1 |
| yellow | UBTF |
| red | UBXN11 |
| blue | UBXN2A |
| green | UBXN6 |
| green | UBXN8 |
| grey | UCHL1 |
| blue | UCK2 |
| blue | UCP2 |
| green | UFM1 |
| grey | UGT1A6 |
| red | UHRF2 |
| blue | ULBP2 |
| grey | ULK1 |
| pink | UNC13D |
| green | UNC45A |
| green | UNC5B |
| grey | UNG |
| blue | UNKL |
| red | UNK |
| green | UPF1 |
| red | UPF3A |
| grey | UPK1B |
| brown | UQCC |
| green | UQCR11 |
| green | UQCRC2 |
| green | USE1 |
| blue | USH1G |
| magenta | USP11 |
| grey | USP21 |
| green | USP27X |
| brown | USP39 |
| blue | USP43 |
| grey | USP5 |
| grey | USP9Y |
| blue | USPL1 |
| green | UTP18 |
| yellow | UXS1 |
| blue | VAMP1 |
| magenta | VAMP3 |
| grey | VAMP4 |
| pink | VAMP5 |
| green | VANGL2 |
| brown | VAPA |
| blue | VASH1 |
| yellow | VASH2 |
| brown | VASN |
| blue | VAV1 |
| yellow | VAV2 |
| yellow | VCAM1 |
| brown | VCAN |
| grey | VCP |
| green | VDAC1 |
| green | VDAC3 |
| green | VEGFA |
| brown | VEGFC |
| brown | VGLL3 |
| grey | VILL |
| pink | VIM |
| magenta | VNN1 |
| blue | VNN2 |
| pink | VOPP1 |
| blue | VPS11 |
| grey | VPS26B |
| magenta | VPS37B |
| grey | VPS37C |
| yellow | VRK2 |
| red | VSIG10 |
| pink | VSIG4 |
| yellow | VSTM2L |
| blue | VTCN1 |
| brown | VWA5A |
| brown | VWF |
| grey | WARS2 |
| red | WASH7P |
| blue | WAS |
| blue | WBP5 |
| yellow | WBSCR17 |
| blue | WDFY4 |
| green | WDR18 |
| yellow | WDR19 |
| red | WDR27 |
| grey | WDR33 |
| blue | WDR41 |
| blue | WDR45L |
| red | WDR62 |
| blue | WDR72 |
| red | WDR73 |
| grey | WDR75 |
| blue | WDR81 |
| red | WDR85 |
| red | WDR90 |
| yellow | WDR91 |
| brown | WDSUB1 |
| grey | WFDC2 |
| brown | WFS1 |
| blue | WHAMM |
| brown | WHSC1 |
| brown | WHSC2 |
| blue | WIPF1 |
| brown | WIPI1 |
| brown | WISP1 |
| yellow | WNK2 |
| yellow | WNT10A |
| yellow | WNT10B |
| yellow | WNT2B |
| brown | WNT2 |
| pink | WNT3A |
| yellow | WNT4 |
| brown | WNT5A |
| yellow | WNT5B |
| brown | WRNIP1 |
| red | WSB1 |
| red | WSB2 |
| yellow | WSCD1 |
| grey | WTAP |
| blue | WWC1 |
| green | WWC2 |
| green | WWC3 |
| yellow | WWOX |
| green | XAB2 |
| blue | XBP1 |
| yellow | XG |
| grey | XK |
| blue | XPC |
| yellow | XPNPEP1 |
| grey | YAF2 |
| green | YARS2 |
| green | YBX1 |
| brown | YBX2 |
| brown | YEATS4 |
| green | YIPF2 |
| grey | YIPF4 |
| red | YJEFN3 |
| pink | YOD1 |
| green | YPEL2 |
| grey | YRDC |
| blue | YWHAQ |
| blue | YWHAZ |
| blue | ZAP70 |
| yellow | ZBED1 |
| blue | ZBP1 |
| blue | ZBTB24 |
| green | ZBTB3 |
| grey | ZBTB42 |
| green | ZBTB45 |
| yellow | ZBTB46 |
| red | ZBTB49 |
| magenta | ZBTB7B |
| brown | ZC3H8 |
| brown | ZCCHC10 |
| brown | ZCCHC24 |
| grey | ZCCHC7 |
| brown | ZCCHC9 |
| brown | ZDHHC13 |
| blue | ZDHHC1 |
| brown | ZDHHC23 |
| brown | ZDHHC2 |
| brown | ZDHHC6 |
| yellow | ZDHHC9 |
| brown | ZEB1 |
| brown | ZEB2 |
| grey | ZFAND1 |
| yellow | ZFP112 |
| grey | ZFP36L2 |
| green | ZFPM1 |
| green | ZFPM2 |
| brown | ZFR2 |
| blue | ZFYVE28 |
| grey | ZFY |
| magenta | ZG16B |
| yellow | ZMIZ2 |
| blue | ZNF101 |
| green | ZNF117 |
| blue | ZNF14 |
| grey | ZNF155 |
| magenta | ZNF165 |
| green | ZNF175 |
| magenta | ZNF185 |
| blue | ZNF187 |
| blue | ZNF211 |
| brown | ZNF234 |
| blue | ZNF235 |
| yellow | ZNF238 |
| red | ZNF248 |
| magenta | ZNF251 |
| blue | ZNF253 |
| grey | ZNF256 |
| green | ZNF25 |
| green | ZNF263 |
| red | ZNF266 |
| grey | ZNF271 |
| grey | ZNF273 |
| brown | ZNF274 |
| red | ZNF276 |
| green | ZNF282 |
| brown | ZNF287 |
| blue | ZNF2 |
| blue | ZNF300 |
| magenta | ZNF323 |
| grey | ZNF324 |
| grey | ZNF329 |
| green | ZNF330 |
| red | ZNF335 |
| red | ZNF337 |
| green | ZNF341 |
| green | ZNF343 |
| blue | ZNF350 |
| green | ZNF358 |
| grey | ZNF362 |
| blue | ZNF383 |
| grey | ZNF385A |
| green | ZNF397OS |
| green | ZNF3 |
| green | ZNF414 |
| blue | ZNF416 |
| blue | ZNF419 |
| brown | ZNF423 |
| grey | ZNF425 |
| grey | ZNF438 |
| blue | ZNF43 |
| blue | ZNF441 |
| grey | ZNF443 |
| brown | ZNF467 |
| brown | ZNF469 |
| green | ZNF480 |
| yellow | ZNF488 |
| green | ZNF48 |
| blue | ZNF490 |
| blue | ZNF502 |
| grey | ZNF503 |
| blue | ZNF506 |
| grey | ZNF512B |
| yellow | ZNF512 |
| red | ZNF513 |
| brown | ZNF521 |
| blue | ZNF526 |
| blue | ZNF527 |
| green | ZNF528 |
| grey | ZNF529 |
| brown | ZNF541 |
| brown | ZNF542 |
| grey | ZNF544 |
| blue | ZNF549 |
| brown | ZNF552 |
| green | ZNF554 |
| blue | ZNF557 |
| green | ZNF564 |
| blue | ZNF566 |
| blue | ZNF569 |
| green | ZNF574 |
| blue | ZNF577 |
| brown | ZNF57 |
| blue | ZNF585A |
| grey | ZNF586 |
| brown | ZNF595 |
| green | ZNF598 |
| blue | ZNF600 |
| blue | ZNF607 |
| grey | ZNF613 |
| green | ZNF628 |
| grey | ZNF629 |
| green | ZNF638 |
| green | ZNF653 |
| brown | ZNF675 |
| blue | ZNF683 |
| red | ZNF692 |
| red | ZNF700 |
| green | ZNF706 |
| grey | ZNF711 |
| brown | ZNF721 |
| blue | ZNF738 |
| blue | ZNF74 |
| magenta | ZNF750 |
| grey | ZNF764 |
| grey | ZNF766 |
| red | ZNF767 |
| green | ZNF777 |
| brown | ZNF77 |
| red | ZNF785 |
| green | ZNF787 |
| red | ZNF789 |
| blue | ZNF793 |
| grey | ZNF799 |
| blue | ZNF79 |
| blue | ZNF814 |
| brown | ZNF823 |
| brown | ZNF830 |
| blue | ZNF831 |
| red | ZNF839 |
| red | ZNF83 |
| green | ZNF841 |
| brown | ZNF853 |
| blue | ZNF879 |
| green | ZNRF2 |
| brown | ZSCAN16 |
| blue | ZSCAN18 |
| green | ZSCAN5A |
| grey | ZW10 |
| brown | ZWINT |
| brown | ZYG11A |
| pink | ZYX |
