# Supplementary material for: NF-κB over-activation portends improved outcomes in HPV-associated head and neck cancer
Source: Oncotarget. 2022 May 24;13:707–22. doi: 10.18632/oncotarget.28232 (PMC9131933; doi:10.18632/oncotarget.28232)
Supplement: Supplementary file 3 [file oncotarget-13-28232-s003.docx]

**Supplementary Table 3. Hypergeometric enrichment analysis comparing WGCNA modules and MISigDB Hallmark Gene Sets**

| Description | p.adjust_blue | p.adjust_brown | p.adjust_yellow | p.adjust_green | p.adjust_red | p.adjust_pink | p.adjust_magenta | Ratio_blue | Ratio_brown | Ratio_yellow | Ratio_green | Ratio_red | Ratio_pink | Ratio_magenta |
| --- | --- | --- | --- | --- | --- | --- | --- | --- | --- | --- | --- | --- | --- | --- |
| HALLMARK_ALLOGRAFT_REJECTION | 1.76E-15 |  |  |  |  | 0.079137123 |  | 0.062937063 |  |  |  |  | 0.052631579 |  |
| HALLMARK_INTERFERON_GAMMA_RESPONSE | 1.42E-06 |  |  |  |  | 0.07236822 |  | 0.037962038 |  |  |  |  | 0.042105263 |  |
| HALLMARK_IL2_STAT5_SIGNALING | 5.27E-05 |  |  |  |  |  |  | 0.041958042 |  |  |  |  |  |  |
| HALLMARK_IL6_JAK_STAT3_SIGNALING | 9.10E-04 |  |  |  |  |  |  | 0.021978022 |  |  |  |  |  |  |
| HALLMARK_INTERFERON_ALPHA_RESPONSE | 0.021450565 |  |  |  |  |  |  | 0.011988012 |  |  |  |  |  |  |
| HALLMARK_INFLAMMATORY_RESPONSE | 0.045898895 |  |  |  |  | 0.007711303 |  | 0.032967033 |  |  |  |  | 0.059649123 |  |
| HALLMARK_COMPLEMENT | 0.074558502 |  |  |  |  | 0.007310177 |  | 0.031968032 |  |  |  |  | 0.063157895 |  |
| HALLMARK_EPITHELIAL_MESENCHYMAL_TRANSITION |  | 1.14E-27 |  |  |  |  |  |  | 0.095472441 |  |  |  |  |  |
| HALLMARK_MYOGENESIS |  | 1.18E-05 |  |  |  |  |  |  | 0.034448819 |  |  |  |  |  |
| HALLMARK_G2M_CHECKPOINT |  | 4.30E-04 |  |  |  |  |  |  | 0.026574803 |  |  |  |  |  |
| HALLMARK_UV_RESPONSE_DN |  | 0.001260384 |  |  |  |  |  |  | 0.027559055 |  |  |  |  |  |
| HALLMARK_E2F_TARGETS |  | 0.002777859 |  |  |  |  |  |  | 0.025590551 |  |  |  |  |  |
| HALLMARK_COAGULATION |  | 0.003516345 |  |  |  | 0.007711303 |  |  | 0.027559055 |  |  |  | 0.045614035 |  |
| HALLMARK_SPERMATOGENESIS |  | 0.015621422 |  |  |  |  |  |  | 0.012795276 |  |  |  |  |  |
| HALLMARK_ANGIOGENESIS |  | 0.030417909 |  |  |  |  |  |  | 0.011811024 |  |  |  |  |  |
| HALLMARK_APICAL_JUNCTION |  | 0.031449798 |  |  |  |  |  |  | 0.030511811 |  |  |  |  |  |
| HALLMARK_TNFA_SIGNALING_VIA_NFKB |  |  | 6.30E-05 |  |  |  |  |  |  | 0.05372617 |  |  |  |  |
| HALLMARK_ESTROGEN_RESPONSE_EARLY |  |  | 0.090145771 |  |  |  |  |  |  | 0.036395147 |  |  |  |  |
| HALLMARK_OXIDATIVE_PHOSPHORYLATION |  |  |  | 1.07E-13 |  |  |  |  |  |  | 0.041666667 |  |  |  |
| HALLMARK_MYC_TARGETS_V1 |  |  |  | 3.38E-07 |  |  |  |  |  |  | 0.028846154 |  |  |  |
| HALLMARK_MYC_TARGETS_V2 |  |  |  | 7.43E-04 |  |  |  |  |  |  | 0.016025641 |  |  |  |
| HALLMARK_ADIPOGENESIS |  |  |  | 0.006594435 |  |  |  |  |  |  | 0.02724359 |  |  |  |
| HALLMARK_DNA_REPAIR |  |  |  |  | 0.097963427 |  |  |  |  |  |  | 0.013623978 |  |  |
| HALLMARK_UNFOLDED_PROTEIN_RESPONSE |  |  |  |  | 0.097963427 |  |  |  |  |  |  | 0.016348774 |  |  |
| HALLMARK_KRAS_SIGNALING_UP |  |  |  |  |  | 0.026587786 |  |  |  |  |  |  | 0.056140351 |  |
| HALLMARK_ESTROGEN_RESPONSE_LATE |  |  |  |  |  |  | 0.005438206 |  |  |  |  |  |  | 0.049 |
| HALLMARK_KRAS_SIGNALING_DN |  |  |  |  |  |  | 0.057049685 |  |  |  |  |  |  | 0.028 |
| HALLMARK_P53_PATHWAY |  |  |  |  |  |  | 0.057049685 |  |  |  |  |  |  | 0.038 |
| Supplemental Table 3. Hypergeometric enrichment analysis comparing WGCNA modules and MISigDB Hallmark Gene Sets. Adjusted P-values are as produced from EnrichR R package. Ratio represents the number of Hallmark gene set genes are members of the indicated WGCNA module. | | | | | | | | | | | | | |  |

Adjusted *P*-values are as produced from EnrichR R package. Ratio represents the number of Hallmark gene set genes are members of the indicated WGCNA module.
