# Supplementary material for: NF-κB over-activation portends improved outcomes in HPV-associated head and neck cancer
Source: Oncotarget. 2022 May 24;13:707–22. doi: 10.18632/oncotarget.28232 (PMC9131933; doi:10.18632/oncotarget.28232)
Supplement: Supplementary file 4 [file oncotarget-13-28232-s004.docx]

**Supplementary Table 5: Alterations in the NF-κB pathway found in the HPV+ TCGA cohort**
